# Characterization by Quantitative Serum Proteomics of Immune-Related Prognostic Biomarkers for COVID-19 Symptomatology

**DOI:** 10.3389/fimmu.2021.730710

**Published:** 2021-09-08

**Authors:** Margarita Villar, José Miguel Urra, Francisco J. Rodríguez-del-Río, Sara Artigas-Jerónimo, Natalia Jiménez-Collados, Elisa Ferreras-Colino, Marinela Contreras, Isabel G. Fernández de Mera, Agustín Estrada-Peña, Christian Gortázar, José de la Fuente

**Affiliations:** ^1^SaBio, Instituto de Investigación en Recursos Cinegéticos IREC-CSIC-UCLM-JCCM, Ciudad Real, Spain; ^2^Biochemistry Section, Faculty of Science and Chemical Technologies, and Regional Centre for Biomedical Research, University of Castilla-La Mancha, Ciudad Real, Spain; ^3^Immunology, Hospital General Universitario de Ciudad Real, Ciudad Real, Spain; ^4^Medicine School, Universidad de Castilla la Mancha, Ciudad Real, Spain; ^5^Local Medical Service Horcajo de los Montes, Ciudad Real, Spain; ^6^Interdisciplinary Laboratory of Clinical Analysis, Interlab-UMU, University of Murcia, Murcia, Spain; ^7^Department of Animal Pathology, Faculty of Veterinary Medicine, University of Zaragoza, Zaragoza, Spain; ^8^Department of Veterinary Pathobiology, Center for Veterinary Health Sciences, Oklahoma State University, Stillwater, OK, United States

**Keywords:** COVID-19, proteomic, immunology, biomarker, diagnostic, prognostic, symptomatology

## Abstract

The COVID-19 pandemic caused by SARS-CoV-2 challenges the understanding of factors affecting disease progression and severity. The identification of prognostic biomarkers and physiological processes associated with disease symptoms is relevant for the development of new diagnostic and therapeutic interventions to contribute to the control of this pandemic. To address this challenge, in this study, we used a quantitative proteomics together with multiple data analysis algorithms to characterize serum protein profiles in five cohorts from healthy to SARS-CoV-2-infected recovered (hospital discharge), nonsevere (hospitalized), and severe [at the intensive care unit (ICU)] cases with increasing systemic inflammation in comparison with healthy individuals sampled prior to the COVID-19 pandemic. The results showed significantly dysregulated proteins and associated biological processes and disorders associated to COVID-19. These results corroborated previous findings in COVID-19 studies and highlighted how the representation of dysregulated serum proteins and associated BPs increases with COVID-19 disease symptomatology from asymptomatic to severe cases. The analysis was then focused on novel disease processes and biomarkers that were correlated with disease symptomatology. To contribute to translational medicine, results corroborated the predictive value of selected immune-related biomarkers for disease recovery [Selenoprotein P (SELENOP) and Serum paraoxonase/arylesterase 1 (PON1)], severity [Carboxypeptidase B2 (CBP2)], and symptomatology [Pregnancy zone protein (PZP)] using protein-specific ELISA tests. Our results contributed to the characterization of SARS-CoV-2–host molecular interactions with potential contributions to the monitoring and control of this pandemic by using immune-related biomarkers associated with disease symptomatology.

## Introduction

Coronavirus disease 19 (COVID-19) is a pandemic caused by severe acute respiratory syndrome coronavirus 2 (SARS-CoV-2, also referred as hCoV-19) with immunological dysregulation associated with disease severity (1, 2). The incidence of this pandemic is still increasing worldwide and posts a challenge for the understanding of host and virus-derived factors affecting disease severity and the identification of prognostic biomarkers and physiological processes related to COVID-19 symptomatology and relevant for the development of new diagnostic and therapeutic interventions to contribute to the control of this pandemic (3–6).

To address this challenge, proteomics constitutes a high-resolution method for the study of host response to infectious diseases, including those caused by RNA viruses (7). Quantitative proteomics has been used for the study of SARS-CoV-2 infection in various samples (e.g., serum, plasma or urine), tissues (e.g., lung), and cells (e.g., peripheral blood mononuclear or Caco-2 cells). This experimental approach has been used for the study of host anti-viral responses and the identification of biomarkers for COVID-19 disease severity, diagnostics, and treatment. Examples of these biomarkers are serum amyloid A-1 (SAA1), serum amyloid A-2 (SAA2), C-reactive protein (CRP), gelsolin (GSN), interleukins (IL-1, IL-6), serine protease inhibitors (SERPINs), progranulin (GRN), apolipoproteins (APOs), complement and pro-inflammatory factors, coagulation system, and vascular cell adhesion protein 1 (VCAM-1) (4–23). Results of proteomics analyses have shown a correlation of disease severity with inflammatory, immunological, and cancer biomarkers, metabolic suppression, neutrophil activation, hepatic and lung injury and the dysregulation of lipid transport, macrophages, platelet degranulation, and complement system pathways (4–6, 8–13, 18, 20–22, 24).

However, due to the complexity of COVID-19 symptomatology, it is important to characterize host response to SARS-CoV-2 infection in different cohorts from asymptomatic individuals to severe patients to better understand disease mechanisms and symptoms with possible medical complications at different levels, and the identification of potential diagnostic markers and drug targets (22–25). Quantitative proteomics approaches alone or in combination with other omics technologies are key to achieve this goal (24–27). To contribute in addressing this objective, herein we used a sequential window acquisition of all theoretical mass spectra (SWATH-MS) quantitative proteomics to characterize serum protein profiles in five cohorts of healthy (pre-pandemic sampling) and SARS-CoV-2-infected asymptomatic, recovered (hospital discharge), nonsevere (hospitalized), and severe [intensive care unit (ICU)] individuals. The results advanced our understanding of the molecular mechanism-driven host–SARS-CoV-2 interactions and identified immune-related prognostic biomarkers and physiological processes related to COVID-19 symptomatology.

## Materials and Methods

### Samples From Healthy Individuals and COVID-19 Patients

**Figure 1:**
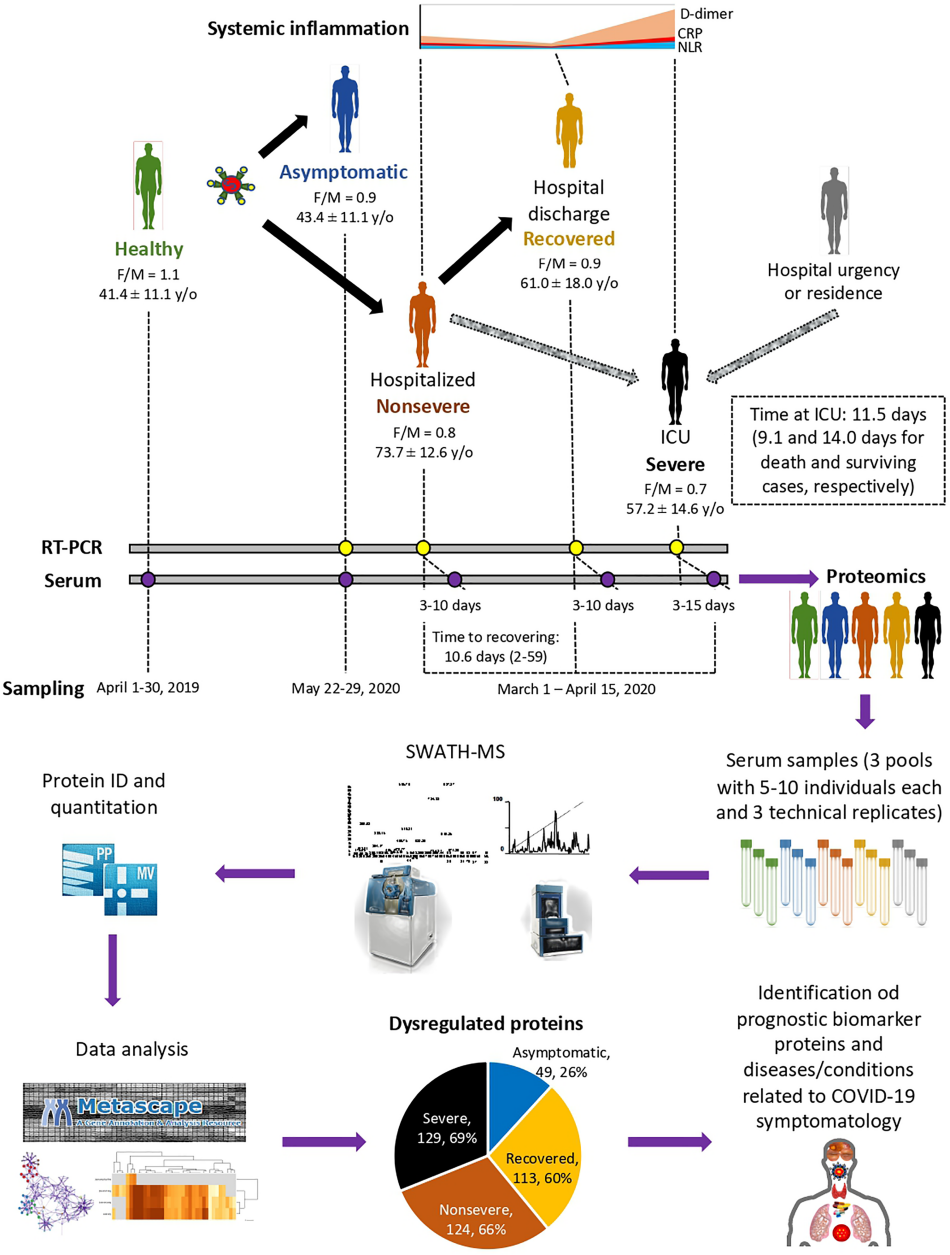
Individual cohorts and study design. COVID-19 patients included cohorts of asymptomatic (*n* = 16), recovered (hospital discharge; *n* = 26), nonsevere (hospitalized; *n* = 28), and severe (ICU; *n* = 25) cases with increasing systemic inflammation. Healthy individuals sampled before the COVID-19 pandemic were included in the analysis (*n* = 25). Female-to-male (F/M) ratio and average ± S.D. age (y/o) are shown. Additional information can be found in Urra et al. (28). A SWATH-MS proteomics approach was used for data acquisition and analysis. A retrospective case–control study was conducted in patients suffering from COVID-19 and healthy controls sampled at indicated dates using standard procedures. Serum from three pools of 5–10 individuals each with three technical replicates were used for proteomics using SWATH-MS protein identification and quantitation and data analysis using Metascape and networks of interactions between proteins and BPs using Graph Theory algorithms to identify dysregulated proteins in response to COVID-19.

A retrospective case–control study was conducted in patients suffering from COVID-19 and healthy controls sampled at the University General Hospital of Ciudad Real (HGUCR), Spain (28, 29). Blood samples from control individuals were collected prior to the COVID-19 pandemic in April 2019. COVID-19 patients were confirmed as SARS-CoV-2-infected by IgG antibody titers or reverse transcriptase-polymerase chain reaction (RT-PCR) and sampled between March and May 2020 (28) (**Figure 1**). Clinical symptoms and laboratory determinations associated with COVID-19 were obtained from patient’s medical records to create cohorts of asymptomatic, nonsevere (hospitalized), recovered (hospital discharge), and severe (ICU) individuals (28). Patients were hospitalized for developing a moderate-severe clinical condition with radiologically demonstrated pneumonia and failure in blood oxygen saturation. Patients with acute respiratory failure who needed mechanical ventilation support were admitted to a hospital ICU. The patients were discharged from the hospital due to the clinical and radiological improvement of pneumonia caused by the SARS-CoV-2, along with the normalization of analytical parameters indicative of inflammation. Data can be found at Urra et al. (28) and **Supplementary Table 1**. Blood samples were drawn in a vacutainer tube without anticoagulant. The tube remained at rest for 15–30 min at room temperature (RT) for clotting. Subsequently, the tube was centrifuged at 1500 × *g* for 10 min at RT to remove the clot and obtain serum. Serum samples were heat-inactivated for 30 min at 56°C and conserved at −20°C until used for analysis. The use of samples and individual data was approved by the Ethical and Scientific Committees (University Hospital of Ciudad Real C-352 and SESCAM C-73).

### Serum Proteomics

Serum samples from healthy controls (*n* = 25) and asymptomatic (*n* = 16), nonsevere (*n* = 28), recovered (*n* = 26), and severe (*n* = 25) COVID-19 individuals were randomly clustered in three biological pools per group (*n* = 5–10 samples per pool). Protein concentration in samples was determined using the BCA Protein Assay with BSA (Sigma-Aldrich) as standard. Protein serum samples (100 µg per sample) were trypsin digested using the FASP Protein Digestion Kit (Expedeon Ltd., UK) and sequencing grade trypsin (Promega, Madison, WI, USA) following the manufacturer’s recommendations. The resulting tryptic peptides were desalted onto OMIX Pipette tips C18 (Agilent Technologies, Santa Clara, CA, USA), dried down, and stored at −20°C until mass spectrometry analysis. The desalted protein digests were resuspended in 2% acetonitrile and 5% acetic acid in water and analyzed by reverse-phase liquid chromatography coupled to mass spectrometry (RP-LC-MS/MS) using an Ekspert™ nanoLC 415 system coupled online with a 6600 TripleTOF mass spectrometer (AB SCIEX; Framingham, US) through Information-Dependent Acquisition (IDA) followed by Sequential Windowed data independent Acquisition of the Total High-resolution Mass Spectra (SWATH-MS). The peptides were concentrated in a 0.1 × 20 mm C18 RP precolumn (Thermo Scientific) with a flow rate of 5 µl/min during 10 min in solvent A. Then, peptides were separated in a 0.075 × 250 mm C18 RP column (New Objective, Woburn, MA, USA) with a flow rate of 300 nl/min. Elution was done in a 120-min gradient from 5% B to 30% B followed by a 15-min gradient from 30% B to 60% B (Solvent A: 0.1% formic acid in water, solvent B: 0.1% formic acid in acetonitrile) and directly injected into the mass spectrometer for analysis.

For IDA experiments, the mass spectrometer was set to scan full spectra from 350 m/z to 1400 m/z (250 ms accumulation time) followed by up to 50 MS/MS scans (100–1500 m/z). Candidate ions with a charge state between +2 and +5 and counts per second above a minimum threshold of 100 were isolated for fragmentation. One MS/MS spectrum was collected for 100 ms, before adding those precursor ions to the exclusion list for 15 s (mass spectrometer operated by Analyst TF 1.6, ABSciex). Dynamic background subtraction was turned off. Data were acquired in high sensitivity mode with rolling collision energy on and a collision energy spread of 5. A total amount of 4 µg of total proteins was injected.

For SWATH quantitative analysis, 45 independent samples (three technical replicates from each of the three biological replicates for each of the five experimental groups) (8 μg each) were subjected to the cyclic data independent acquisition (DIA) of mass spectra using the SWATH variable windows calculator (V 1.0, AB SCIEX) and the SWATH acquisition method editor (AB SCIEX), similar to established methods (30). A set of 50 overlapping windows was constructed (containing 1 m/z for the window overlap), covering the precursor mass range of 400–1250 m/z. For these experiments, a 50-ms survey scan (350–1400 m/z) was acquired at the beginning of each cycle, and SWATH-MS/MS spectra were collected from 100 to 1500 m/z for 70 ms at high sensitivity mode, resulting in a cycle time of 3.6 s. Collision energy for each window was determined according to the calculation for a charge +2 ion-centered upon the window with a collision energy spread of 15.

To create a spectral library of all the detectable peptides in the samples, the IDA MS raw files were combined and subjected to database searches in unison using ProteinPilot software v. 5.0.1 (AB SCIEX) with the Paragon algorithm. Spectra identification was performed by searching against the UniProt human proteome database (75,074 entries in October 2020) with the following parameters: iodoacetamide cysteine alkylation, trypsin digestion, identification focus on biological modification, and thorough ID as search effort. The detected protein threshold was set at 0.05. To assess the quality of identifications, an independent False Discovery Rate (FDR) analysis with the target-decoy approach provided by Protein Pilot was performed. Positive identifications were considered when identified proteins reached a 1% global FDR.

The mass spectrometry proteomics data have been deposited to the ProteomeXchange Consortium *via* the PRIDE (31) partner repository with the dataset identifier PXD024549 and 10.6019/PXD024549.

### Quality Control of Proteomics Data

Quality of proteomics data was controlled at multiple levels. First, a rat ileum digest was used for the evaluation of instrument performance. Buffer A samples were run as blanks every three injections to prevent carryover. Three technical replicates were injected for each sample. For validation of serum proteomics data, protein representation for previously identified biomarkers for COVID-19 and proteomics studies were used to show correlation with disease severity (**Supplementary Figures 3**, **4**). An enrichment analysis was conducted using the Coronascape COVID database (https://metascape.org/COVID) (32) to identify proteins found in our study as differentially represented in response to COVID-19 and reported in previous COVID-19 omics datasets.

### Data Analysis

For SWATH processing, up to 10 peptides with seven transitions per protein were automatically selected by the SWATH Acquisition MicroApp 2.0 in the PeakView 2.2 software with the following parameters: 15 ppm ion library tolerance, 5 min XIC extraction window, 0.01 Da XIC width, and considering only peptides with at least 99% confidence and excluding those that were shared or contained modifications. However, to ensure reliable quantitation, only proteins with three or more peptides available for quantitation were selected for XIC peak area extraction and exported for analysis in the MarkerView 1.3 software (AB SCIEX). Global normalization according to the Total Area Sums of all detected proteins in the samples was conducted (**Supplementary Data 1**).

The Student’s *t*-test (*p* < 0.05) was used to perform two-sample comparisons between the averaged area sums of all the transitions derived for each protein across the nine replicate runs for each group under comparison, in order to identify proteins that were significantly differentially represented between groups (**Supplementary Data 1**). Protein representation was also compared between groups by Welch’s unpaired *t*-test (*p* < 0.05; https://www.graphpad.com/quickcalcs/ttest1/?Format=C) and by one-way ANOVA test followed by post-hoc Bonferroni and Holm multiple comparisons (*p* < 0.05; https://astatsa.com/OneWay_Anova_with_TukeyHSD/) (4). Proteins with significant differences between healthy individuals and one of the COVID-19 cohorts only were selected for heatmap analysis of *z*-score using complete linkage and Spearman rank correlation (http://www.heatmapper.ca/expression/). Data were separately analyzed for overrepresented and underrepresented proteins using the Metascape gene annotation and analysis resource (https://metascape.org/gp/index.html#/main/step1) (**Supplementary Figure 1**).

To evaluate the network of interactions between proteins and BPs, a network was built using data for each protein and the BPs in which it is involved (**Supplementary Data 2**). This network reflects the importance of each protein on each BP according to its representation. The purpose was to obtain a general framework based on previous network developments using Graph Theory algorithms, which were revealed to be adequate for the purpose of representing these relationships (33). Networks exhibit nodes and the relationships between these components (links). Each node represents a protein or a BP. The network is directed, as each edge links each protein “to” one or multiple BPs. Several indices measure network properties from which the relationships among proteins and BPs are derived. The weighted degree (WG) is one of the most basic measures of a network, representing the number of links leaving (or arriving at) a given node after weighting by the total number of records containing this interaction. In this context, a protein always links to a BP with a “strength” derived from its representation. The WG provides an estimation of the strength of the association but does not evaluate the importance of each node in the context of the network. We used the Page Rank (PR) index to calculate the importance of each node in the complete network (34). This index calculates the number of links of each protein to one or several BPs, together with its weighted degree. The PR of each protein is calculated according to the authority (i.e., the relative importance) of each BP. The PR is an index that assigns a universal rank to nodes based on the importance of the other nodes to which it is linked and the WG. We calculated PR for each cohort (healthy, asymptomatic, recovered, nonsevere, and severe COVID-19 cases) and built separate networks for each condition. Then, we calculated how PR of both proteins and BPs changed in each group. We were looking for prominent changes in the nodes of the network, using an approach based on the distribution of values and the semantic rules of Fuzzy Logic (35). For each node of the network, we selected all the nodes that were in the first quintile (i.e., lowest values) of the PR’s distribution of groups “healthy” and “asymptomatic” and that were in the last quintile (i.e., high values) of distribution of groups “nonsevere” and “severe”. The opposite selection (highest versus lowest) was also carried out. After relating these queries by the operator “AND” according to Fuzzy Logic rules, each node was ranked between 0 (no change) and 1 (maximum change). We arbitrarily removed the nodes with values lower than 0.5. We also evaluated the weighted nestedness of each network as a measure of structuring. A network is more coherent and robust (i.e., resilient to node removal) if structuring is high. Nestedness is a measure in ecological system networks that emanates from the way elements are linked. It should be noted that the absence of nestedness does not mean the absence of a pattern. Nestedness is not a feature of the network, but a consequence of the WD sequences (36). Since most of the available algorithms evaluate the nestedness using only the pattern presence/absence (i.e., interaction/not interaction), we adhered to the approach provided by the software WINE (37) since it also accounts for the weights of the interactions in quantitative data matrices (proteins and BPs in our application) that include the number of events of each interaction and the strength of such interaction, or the representation of the proteins involved in each BP.

### Determination of IL-1 and IL-4 Serum Levels

Serum levels of IL-1 and IL-4 were determined by ELISA (Invitrogen, Carlsbad, CA, USA) following the manufacturer’s instructions. Briefly, 96-microwell plates coated in duplicate with anti-human IL-1β or IL-4 were washed twice with 400 µl/well of wash buffer and 100 µl of human IL-1β or IL-4 standard (20.00 pg/ml) at serial dilutions (1:2, 1:4, 1:8, 1:16, and 1:32), 100 µl/well of sera at 1:2 dilution, and 100 µl/well of sample diluent as negative control. Then, 50 µl/well of biotin conjugate were added to all wells. After incubation for 2 h at RT and three washes with 400 µl/well of wash buffer, 100 µl/well of streptaviding-HRP were added to all wells. After incubation for 1 h at RT and three washes with 400 µl/well of wash buffer, 100 µl/well of 3,3′,5,5′-Tetramethylbenzidine or TMB substrate solution were added to all wells. As soon as the Standard 1 well reached an O.D. of 0.9 at 620 nm, the colorimetric reaction was stopped with 100 µl/well of stop solution and the absorbance was measured in a spectrophotometer (Thermo Fisher Scientific, Waltham, MA, USA) at an O.D. of 450 nm; 0.05 Human IL-1β or IL-4 concentration (pg/ml) in each sample was calculated from the obtained standard curve. The results were compared between different groups by one-way ANOVA test with post-hoc Tukey Honestly Significant Difference (HSD) (https://astatsa.com/OneWay_Anova_with_TukeyHSD/; *p* = 0.05).

### Validation of Selected Serum Protein Biomarkers

Serum samples from cohorts included in the proteomics analysis plus additional samples of healthy controls (*n* = 37) and asymptomatic (*n* = 18), nonsevere (*n* = 29), recovered (*n* = 27), and severe (*n* = 25) COVID-19 individuals were used for validation analysis. Serum levels of PZP, SELENOP, CBP2, and PON1 were determined by ELISA (MyBioSource, Inc., San Diego, CA, USA, provided by bioNova Científica S.L., Madrid, Spain) following the manufacturer’s protocol available online (PZP, MBS2706073, https://www.mybiosource.com/human-elisa-kits/pregnancy-zone-protein-pzp/2706073; SELENOP, MBS163893, https://www.mybiosource.com/human-elisa-kits/selenoprotein-p-se-p/163893; CPB2, MBS703133, https://www.mybiosource.com/cpb2-human-elisa-kits/carboxypeptidase-b2-plasma/703133; PON1, MBS2883206, https://www.mybiosource.com/pon1-human-elisa-kits/serum-paraoxonase-arylesterase-1/2883206). The results were compared between different groups by one-way ANOVA test with post-hoc Tukey HSD (https://astatsa.com/OneWay_Anova_with_TukeyHSD/; *p* = 0.05). Proteomics and ELISA data were compared by Spearman’s Rho (rs) correlation analysis (https://www.socscistatistics.com/tests/spearman/default2.aspx; *p* = 0.05).

## Results

### Variations in Differential Serum Protein Profiles and Affected Biological Processes According to COVID-19 Disease Symptomatology

The study was conducted using a SWATH-MS quantitative proteomics to characterize serum protein profiles in COVID-19 patient cohorts from asymptomatic to recovered (hospital discharge), nonsevere (hospitalized), and severe (ICU) cases with increasing systemic inflammation in comparison with healthy individuals sampled prior to the COVID-19 pandemic (**Figure 1**). A total of 189 proteins were identified in serum samples from all cohorts included in the study (**Supplementary Data 1**). Of them, 49, 113, 124, and 129 proteins were significantly dysregulated in asymptomatic, recovered, nonsevere, and severe cases when compared to healthy controls, respectively (**Figure 1**; **Supplementary Figure 1** and **Data 1**). As expected, immunoglobulins, high-density lipoproteins (HDL) and complement cascade represented 32% (60/189), 23% (44/189), and 12% (22/189) of identified serum proteins, respectively (**Supplementary Data 1**).

**Figure 2:**
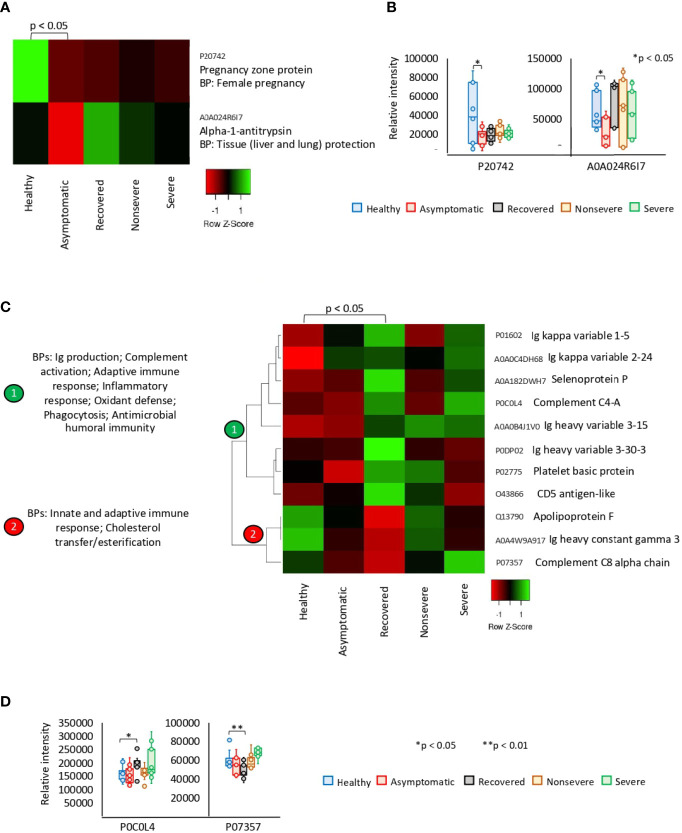
Exclusive differential representation of proteins in sera from COVID-19 asymptomatic and recovered cases. **(A)** Heatmap of proteins significantly dysregulated (*Z*-scored original value) in asymptomatic cases only (*p* < 0.05; unpaired two-sided Welch’s *t*-test). Biological process (BP) is shown for each protein. **(B)** Change in levels of two selected proteins with significant differences between asymptomatic cases and healthy controls (*p < 0.05; unpaired two-sided Welch’s *t*-test). **(C)** Heatmap of proteins significantly dysregulated (*Z*-scored original value) in recovered cases only (*p* < 0.05; unpaired two-sided Welch’s *t*-test). Biological processes (BPs) are shown for each cluster of proteins differentially represented in response to COVID-19 (cluster 1, overrepresented; cluster 2, underrepresented). **(D)** Change in levels of two selected proteins with significant differences between recovered cases and healthy controls (*p < 0.05, **p < 0.01; unpaired two-sided Welch’s *t*-test).

Of the significantly dysregulated proteins, Pregnancy zone protein (PZP) and Alpha-1-antitrypsin (SERPINA1) were identified as underrepresented in asymptomatic cases only (**Figures 2A, B**). These proteins are involved in biological processes (BPs) of female pregnancy and tissue protection. In recovered COVID-19 cases, 11 proteins were exclusively significantly dysregulated and grouped in two clades of overrepresented (*n* = 8) and underrepresented (*n* = 3) proteins (**Figures 2C, D**). Patient’s recovery was associated with dysregulation of immune response; increased complement activation, inflammatory response, and oxidant defense; and decrease in cholesterol transfer/esterification.

**Figure 3:**
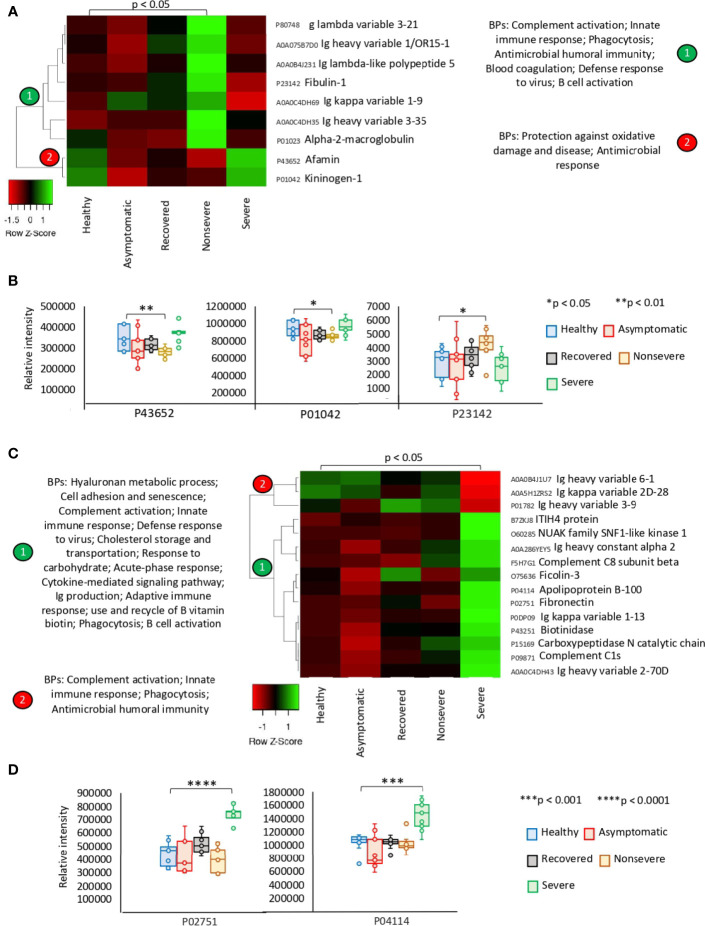
Exclusive differential representation of proteins in sera from COVID-19 nonsevere and severe cases. **(A)** Heatmap of proteins significantly dysregulated (*Z*-scored original value) in nonsevere cases only (*p* < 0.05; unpaired two-sided Welch’s *t*-test). Biological processes (BPs) are shown for each cluster of proteins differentially represented in response to COVID-19 (cluster 1, overrepresented; cluster 2, underrepresented). **(B)** Change in levels of three selected proteins with significant differences between nonsevere cases and healthy controls (*p < 0.05, **p < 0.01; unpaired two-sided Welch’s *t*-test). **(C)** Heatmap of proteins significantly dysregulated (*Z*-scored original value) in severe cases only (*p* < 0.05; unpaired two-sided Welch’s *t*-test). Biological processes (BPs) are shown for each cluster of proteins differentially represented in response to COVID-19 (cluster 1, overrepresented; cluster 2, underrepresented). **(D)** Change in levels of two selected proteins with significant differences between severe cases and healthy controls (***p < 0.001, ****p < 0.0001; unpaired two-sided Welch’s *t*-test).

**Figure 4:**
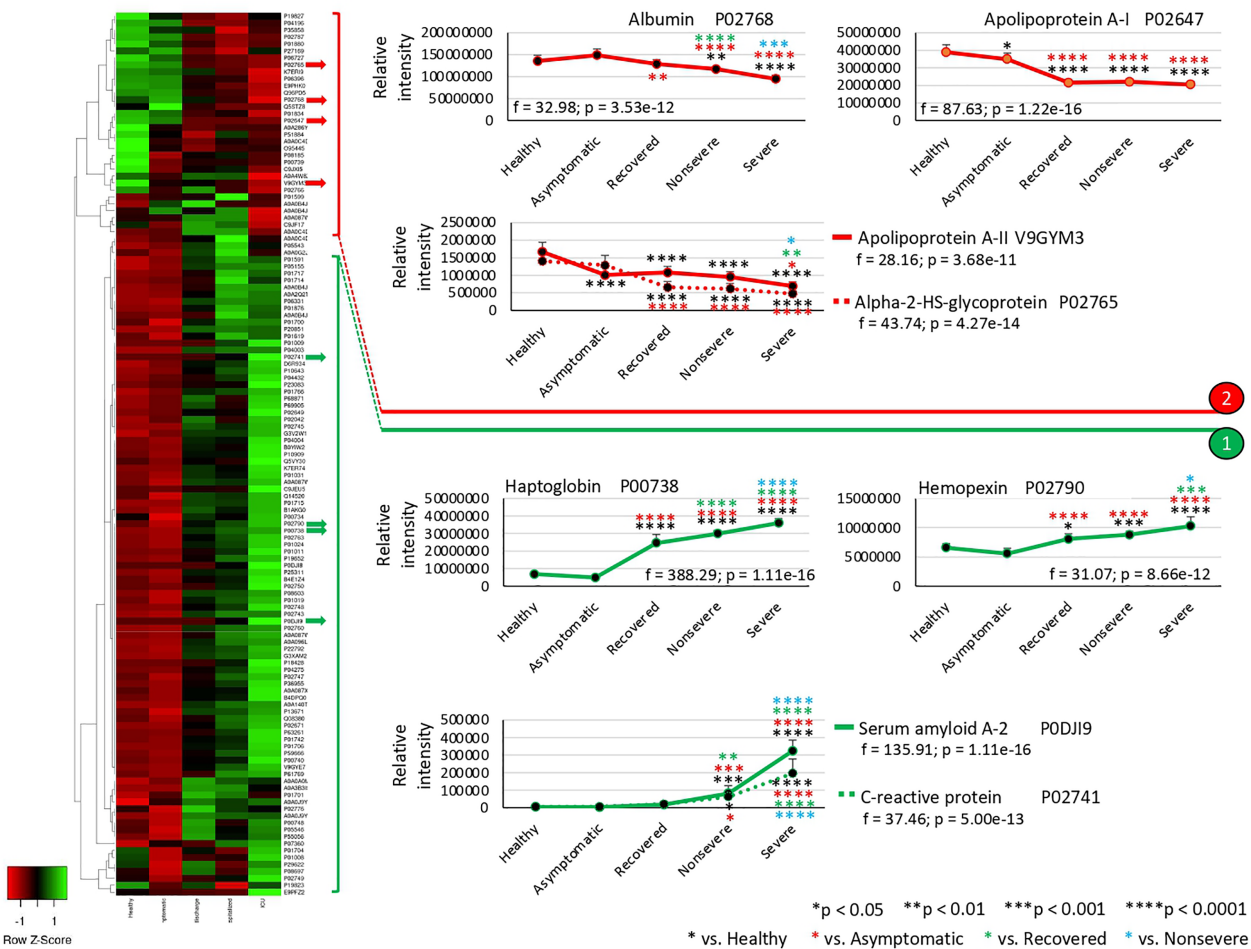
Multiple differential representation of proteins in sera from COVID-19 cases. Heatmap of proteins significantly dysregulated (*Z*-scored original value) in multiple COVID-19 cohorts (*p* < 0.05; unpaired two-sided Welch’s *t*-test). Clusters of proteins differentially represented in response to COVID-19 (cluster 1, overrepresented; cluster 2, underrepresented) are shown. Protein levels of four selected proteins with significant differences on each cluster were compared between groups by one-way ANOVA test followed by post-hoc Bonferroni and Holm multiple comparisons (*f*-values and *p*-values are shown) and unpaired two-sided Welch’s *t*-test (*p < 0.05, **p < 0.01, (***p < 0.001, ****p < 0.0001).

The exclusively significantly dysregulated serum proteins in nonsevere (*n* = 9) and severe (*n* = 15) patients affected multiple BPs (**Figures 3A–D**). In nonsevere cases, overrepresented proteins (*n* = 7) are involved in complement activation, immune response, and blood coagulation while underrepresented proteins (*n* = 2) reduce protection against oxidative damage and disease. Severe cases showed dysregulation of BPs such as immune response, metabolic processes, complement activation, and response to carbohydrate associated with overrepresented proteins (*n* = 12). Exclusively underrepresented proteins in severe cases (*n* = 3) are involved in immune response and complement activation. Proteins with multiple differential representation in sera from COVID-19 cases when compared to healthy controls (*n* = 128) were grouped into two clades of proteins with a tendency towards increase (*n* = 93) and decrease (*n* = 35) in representation according to disease severity (**Figure 4**).

**Figure 5:**
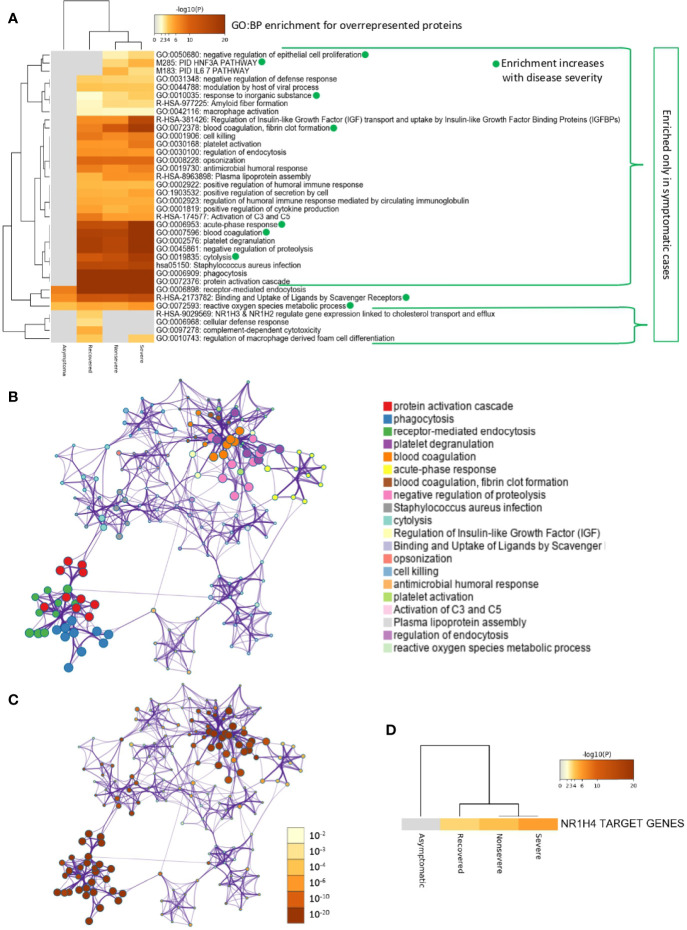
Enrichment ontology clusters for differentially overrepresented proteins in sera from COVID-19 cases. **(A)** Statistically enriched terms (GO/KEGG biological processes; GO : BP). Accumulative hypergeometric *p*-values and enrichment factors were calculated and used for filtering. Remaining significant terms were then hierarchically clustered into a tree based on Kappa-statistical similarities among their protein memberships (as used in DAVID Bioinformatics Resources 6.8; https://david.ncifcrf.gov). A 0.3 Kappa score was applied as the threshold to cast the tree into term clusters. The term with the best *p*-value within each cluster was selected as its representative term and displayed in a dendrogram. The heatmap cells are colored by their *p*-values; white cells indicate the lack of enrichment for that term in the corresponding gene list. BPs in which enrichment increased with disease severity only in symptomatic cases are shown. **(B)** Network of enriched terms. We selected a subset of representative terms from the full cluster and convert them into a network layout. More specifically, each term is represented by a circle node, where its size is proportional to the number of input genes that fall into that term, and its color represents its cluster identity (i.e., nodes of the same color belong to the same cluster). Terms with a similarity score > 0.3 are linked by an edge (the thickness of the edge represents the similarity score). The network is visualized with Cytoscape (v3.1.2) with “force-directed” layout and with edge bundled for clarity. One term from each cluster is selected to have its term description shown as label. **(C)** Network of enriched terms colored by *p*-value. The same enrichment network has its nodes colored by *p*-value, as shown in the legend. The darker the color, the more statistically significant the node is (see legend for *p*-value ranges). **(D)** Quality control and association analysis. Protein lists were identified in the ontology categories Transcription_Factor_Targets. All genes in the genome were used as the enrichment background. Terms with a *p*-value < 0.01, a minimum count of 3, and an enrichment factor (ratio between the observed counts and the counts expected by chance) > 1.5 were collected and grouped into clusters. The algorithm used here is the same as that used in the other enrichment analyses.

**Figure 6:**
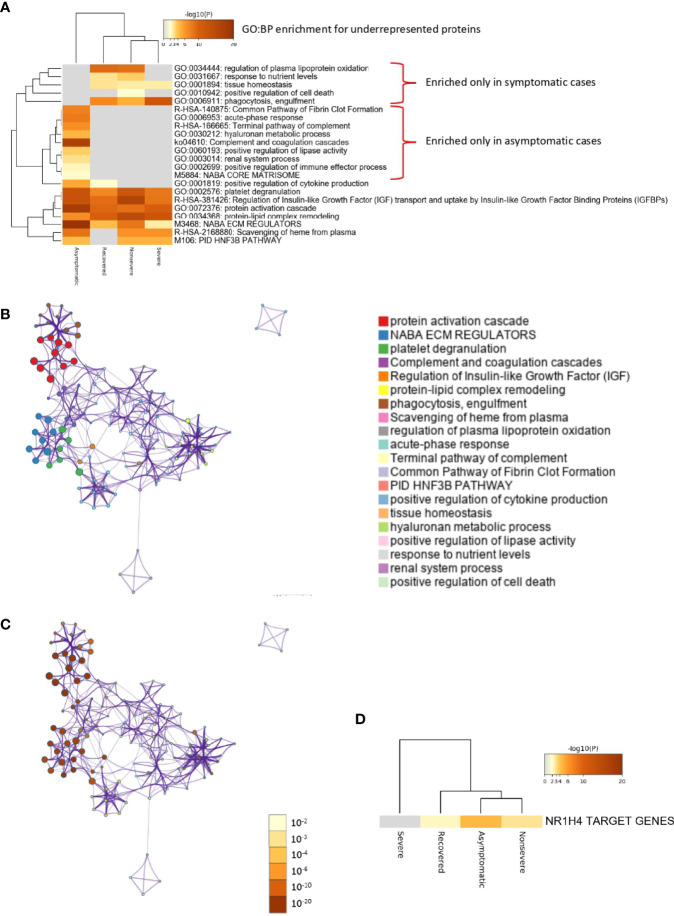
Enrichment ontology clusters for differentially underrepresented proteins in sera from COVID-19 cases. **(A)** Statistically enriched terms (GO/KEGG biological processes; GO : BP). Accumulative hypergeometric *p*-values and enrichment factors were calculated and used for filtering. Remaining significant terms were then hierarchically clustered into a tree based on Kappa-statistical similarities among their protein memberships (as used in DAVID Bioinformatics Resources 6.8; https://david.ncifcrf.gov). A 0.3 Kappa score was applied as the threshold to cast the tree into term clusters. The term with the best *p*-value within each cluster was selected as its representative term and displayed in a dendrogram. The heatmap cells are colored by their *p*-values; white cells indicate the lack of enrichment for that term in the corresponding gene list. BPs enriched only in symptomatic or asymptomatic cases are shown. **(B)** Network of enriched terms. We selected a subset of representative terms from the full cluster and convert them into a network layout. More specifically, each term is represented by a circle node, where its size is proportional to the number of input genes that fall into that term, and its color represents its cluster identity (i.e., nodes of the same color belong to the same cluster). Terms with a similarity score > 0.3 are linked by an edge (the thickness of the edge represents the similarity score). The network is visualized with Cytoscape (v3.1.2) with “force-directed” layout and with edge bundled for clarity. One term from each cluster is selected to have its term description shown as label. **(C)** Network of enriched terms colored by *p*-value. The same enrichment network has its nodes colored by *p*-value, as shown in the legend. The darker the color, the more statistically significant the node is (see legend for *p*-value ranges). **(D)** Quality control and association analysis. Protein lists were identified in the ontology categories Transcription_Factor_Targets. All genes in the genome were used as the enrichment background. Terms with a *p*-value < 0.01, a minimum count of 3, and an enrichment factor (ratio between the observed counts and the counts expected by chance) > 1.5 were collected and grouped into clusters. The algorithm used here is the same as that used in the other enrichment analyses.

Of the multiple BPs affected by significantly dysregulated serum proteins, some were only enriched in symptomatic cases while others were enriched in asymptomatic cases (**Figures 5A–D** and **6A–D**). For overrepresented proteins, enrichment increased with disease severity for BPs such as negative regulation of epithelial cell proliferation, FOXA1 transcription factor network (HNF3A pathway M285) coordinating function of primary airway epithelial cells, IL-6-mediated signaling events (M183), response to inorganic substance, blood coagulation, acute-phase response, cytolysis, binding and uptake of ligands by scavenger receptors, and reactive oxygen species metabolic process (**Figure 5A**). Underrepresented proteins were enriched only in both asymptomatic (e.g., common pathway of fibrin clot formation, acute-phase response, complement and coagulation cascade, hyaluronan metabolic process, positive regulation of lipase activity, renal system process, positive regulation of immune effector process and M5884 ensemble of genes encoding core extracellular matrix including ECM glycoproteins, collagens, and proteoglycans) and symptomatic (e.g., regulation of plasma lipoprotein oxidation, response to nutrient levels, tissue homeostasis, positive regulation of cell death, and phagocytosis) cases (**Figure 6A**).

**Figure 7:**
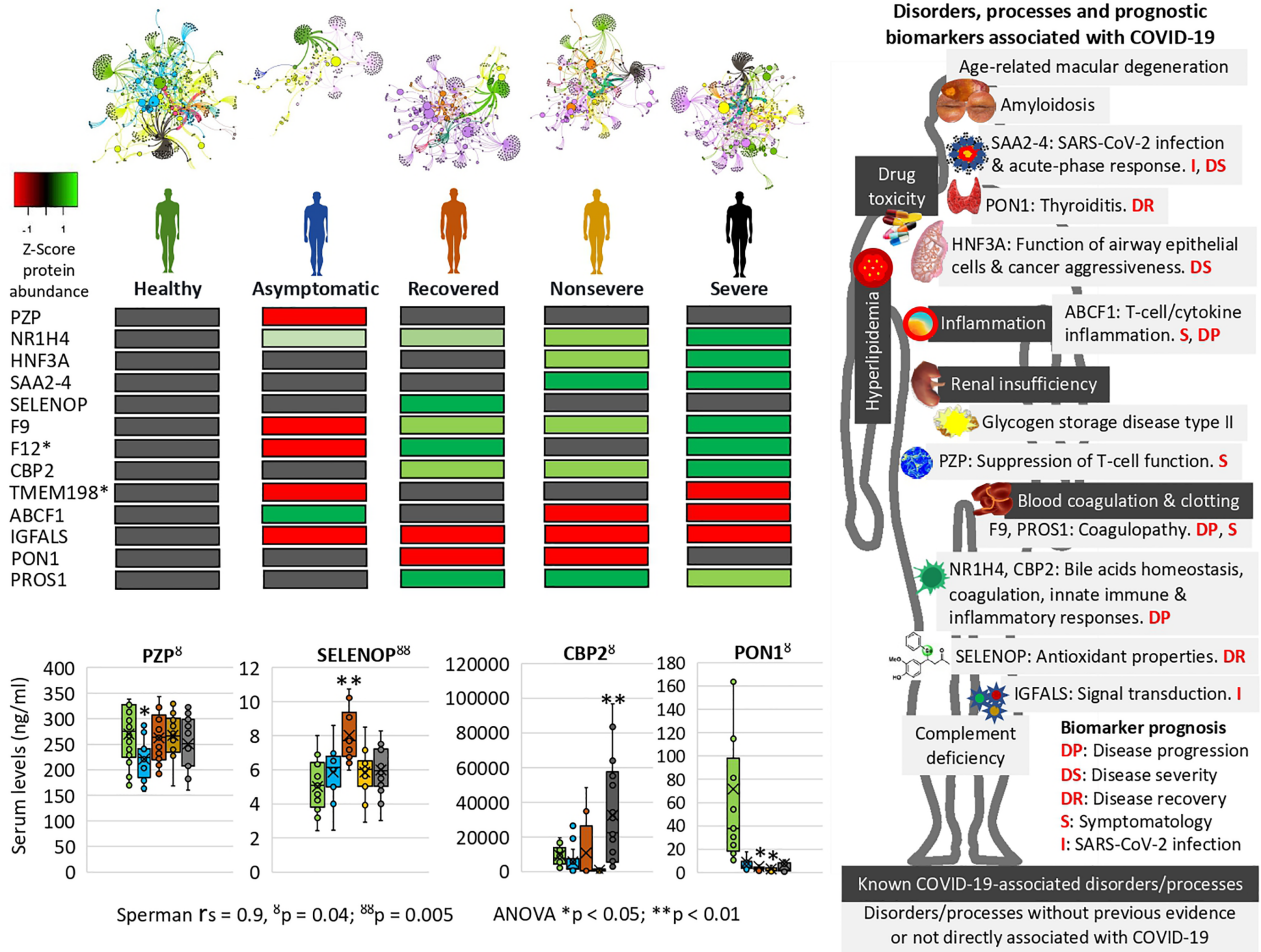
Prognostic biomarker proteins related to COVID-19 symptomatology. Network analysis of interactions between proteins and BPs reflects nestedness or structuring of the cluster’s magnitude decreasing with COVID-19 symptomatology. SWATH-MS quantitative serum proteomics identified proteins involved in physiological disorders and processes associated with COVID-19 and novel biomarker proteins with potential implications for the development of new diagnostic and therapeutic interventions to contribute to the control of this pandemic. *Unsupportive protein profile for prognostic biomarker. Selected serum biomarkers (PZP, SELENOP, CBP2, and PON1) were validated by ELISA. Change in protein serum levels with significant differences in comparison to healthy controls (*p < 0.05, **p < 0.01; one-way ANOVA test with post-hoc Tukey HSD). Proteomics and ELISA data were compared by Spearman’s Rho (rs) correlation analysis (^ŏ^ *p* < 0.05, ^ŏŏ^ *p* < 0.01).

The network of interactions between proteins and BPs was characterized using Graph Theory algorithms (**Supplementary Figure 2** and **Data 2**). While visually similar, networks have deep differences in their structure. Other than obvious changes of the proteins involved (presence/absence and representation), therefore affecting the BPs, nestedness showed a decreasing magnitude according to the patient cohorts. Nestedness is maximum for healthy and asymptomatic individuals (nestedness of 12.2 and 12.1, respectively), which reflects a high structuring of the clusters (**Figure 7** and **Supplementary Data 2**). However, networks built using proteins and BPs for nonsevere and severe patients show a clear de-structuring (nestedness of 5.1 and 3.8, respectively). The networking built with data of recovered patients shows an intermediate structure without clear differences with other cohorts in this analysis (nestedness of 10.1). These results point to a clear pattern in which some proteins (rate of change > 0.900; **Supplementary Data 2**) such as neutrophil defensin 3, serum amyloid A (SAA) SAA2-SAA4 readthrough, Apolipoprotein C-IV, and Fibrinogen gamma chain are associated with nonsevere and severe COVID-19 patients, therefore increasing the PR index value of the BPs. It seems that overrepresentation of selected proteins in patients with higher COVID-19 symptomatology is blocking the normal regulation of these BPs, which resulted in higher PR values in these cohorts. Networks resulting from these cohorts are de-structured, and the structure with clear clusters observed in healthy individuals is not evident. Therefore, the networks produced with proteins and the BPs in the five cohorts show critical changes. These changes include the overrepresentation of some BPs such as negative regulation by a host of viral processes, negative regulation of mononuclear cell proliferation, positive regulation of interleukins, positive regulation of chemokine production, and positive regulation of respiratory burst involved in inflammatory response that remained unaltered in healthy and recovered individuals. These results support the idea that a network construct, based on pure statistical rules, reflects the clinical status commonly observed in critical COVID-19 patients.

In correspondence with these BPs, the network of enriched terms showed that the most represented processes in proteins overrepresented in COVID-19 cohorts are protein activation cascade, phagocytosis, receptor-mediated endocytosis, platelet degranulation, blood coagulation, acute-phase response, negative regulation of proteolysis, *Staphylococcus aureus* infection, cytolysis, regulation of insulin-like growth factor (IGF), binding and uptake of ligands by scavenger, opsonization, cell killing, antimicrobial humoral response, platelet activation, activation of complement C3 and C5, plasma lipoprotein assembly, regulation of endocytosis, and reactive oxygen species metabolic process (**Figures 5B, C**). For underrepresented proteins, the most enriched processes were protein activation cascade, enzymes and their regulators involved in the remodeling of the extracellular matrix (NABA ECM regulators), platelet degranulation, complement and coagulation cascades, regulation of IGF, protein–lipid complex remodeling, phagocytosis, scavenging of heme from plasma, regulation of plasma lipoprotein oxidation, acute-phase response, terminal pathway of complement, pathway of fibrin clot formation, FOXA2 and FOXA3 transcription factor networks (HNF3B pathway M106), positive regulation of cytokine production, tissue homeostasis, hyaluronan metabolic process, positive regulation of lipase activity, response to nutrient levels, renal system process, and positive regulation of cell death (**Figures 6B, C**). The quality control and association analysis showed that network representation of nuclear receptor subfamily 1, group H, member 4 (NR1H4) target genes increased with disease severity (**Figures 5D**, **6D**). Protein–protein interaction enrichment analysis resulted in complement, coagulation, and clotting cascades for overrepresented proteins and lipoprotein particle remodeling, reverse cholesterol transport, and peptide ligand-binding receptors for underrepresented proteins (**Supplementary Figure 1**).

### Identification of Prognostic Biomarkers in Proteins Associated With COVID-19 Disease Symptomatology

For validation of serum proteomics data, an enrichment analysis was conducted using the Coronascape COVID database (https://metascape.org/COVID) to identify proteins found in our study as differentially represented in response to COVID-19 and reported in previous COVID-19 omics datasets as a correlate of disease severity (**Supplementary Figures 3**, **4**). This analysis also identified proteins dysregulated in COVID-19 patients and potentially not previously associated with disease symptomatology (**Supplementary Figure 5**). Of these proteins, several were previously identified as biomarkers of severe COVID-19 in non-omics studies and were not included in further analyses (**Supplementary Figure 5**).

However, other proteins not previously identified in COVID-19 patients or with differences in the representation profile compared to our study were proposed as novel in relation to disease symptomatology and were used for prognostic biomarkers identification (**Table 1** and **Supplementary Figure 5**). Of them, coagulation factor XII (F12) and transmembrane protein 198 (TMEM198) showed an unsupportive profile for biomarker prediction (**Table 1** and **Figure 7**). TMEM198 has been associated with diabetes as observed in comorbidities of COVID-19 symptomatic cohorts included in the study (**Supplementary Table 1**).

**Table 1:** Candidate prognostic biomarker proteins related to COVID-19 disease symptomatology.

Proteins	Results of our study	Previous findings	Biomarker predictor	Refs
Selenoprotein P (SELENOP)	Overrepresented in recovered cases	Lower levels in COVID-19 patients	Disease recovery Validated by ELISA	(38)
Coagulation factor IX (F9)	Overrepresented in all COVID-19 patients. Correlation with symptomatology	Decrease in protein levels from nonsevere to severe patients	Disease progression	(22, 39, 40)
Coagulation factor XII (F12)	Overrepresented in all but nonsevere COVID-19 patients	Not identified	Unsupportive profile	(40)
Carboxypeptidase B2 (CPB2)	Overrepresented in all but asymptomatic COVID-19 patients	Not identified	Disease severity Validated by ELISA	(41)
Transmembrane protein 198 (TMEM198)	Underrepresented in asymptomatic and severe COVID-19 patients	Not identified	Unsupportive profile	(42, 43)
ATP-binding cassette sub-family F member 1 (ABCF1)	Overrepresented in asymptomatic and underrepresented in nonsevere and severe COVID-19 patients	Not identified	Symptomatology and disease progression	(44)
Insulin-like growth factor-binding protein complex acid labile subunit (IGFALS)	Underrepresented in all COVID-19 patients	Increase in protein levels from nonsevere to severe patients	SARS-CoV-2 infection	(22, 44) (45) (46) (47)
Serum paraoxonase/ arylesterase 1 (PON1)	Underrepresented in nonsevere and recovered COVID-19 patients	Increase in protein levels from nonsevere to severe patients	Disease recovery Reduction in thyroiditis Validated by ELISA	(22, 48, 49)
Pregnancy zone protein (PZP)	Underrepresented only in asymptomatic cases	Not identified	Symptomatology Validated by ELISA	(50)
Vitamin K-dependent protein S (PROS1)	Overrepresented in recovered, nonsevere, and severe COVID-19 patients but with lower levels in severe cases	Associated with COVID-19 coagulopathy	Disease progression Symptomatology	(51)

Proteins related to COVID-19 symptomatology and used for the identification of candidate prognosis biomarkers. Protein representation refers to significant differences when compared to healthy controls. Full data are disclosed in **Supplementary Data 1** and **Figures 5**, **7**.

Selected identified candidate prognostic immune-related biomarker proteins, PZP, Selenoprotein P (SELENOP), Carboxypeptidase B2 (CPB2), and Serum paraoxonase/arylesterase 1 (PON1) (**Table 1**), were validated by ELISA using sera from individuals of all cohorts included in the study (**Figure 7**). The results corroborated the predictive value of these biomarkers for disease recovery (SELENOP and PON1), severity (CBP2), and symptomatology (PZP).

### Characterization of Differentially Represented Proteins in Response to COVID-19 and Associated to Other Human Diseases and Conditions to Monitor Risk Factors for Disease Symptomatology

An enrichment analysis was conducted using the DisGeNET discovery platform (https://www.disgenet.org) provided by Metascape (https://metascape.org) to identify proteins differentially represented in response to COVID-19 and associated to other human diseases and conditions with major affected physiological processes resulting in macrophage activation and coagulopathy (**Figure 7** and **Supplementary Figure 6**). The results showed two main types of pathologies enriched with disease severity, renal insufficiency (acute kidney injury, acute kidney insufficiency, proteinuria, and nephrotic syndrome) and blood coagulation alterations (factor V Leiden mutation, activated protein C resistance, and lupus anticoagulant disorder). Alterations in blood coagulation are a consequence of the SARS-CoV-2 infection and the associated pro-inflammatory processes (52, 53). Three of the identified pathologies (factor V Leiden mutation, activated protein C resistance, and lupus anticoagulant disorder) are related to pro-coagulant alterations and have been clinically associated with COVID-19 coagulopathy (54). Renal insufficiency has been associated with poor COVID-19 prognosis (55), and the results correlated with renal disease comorbidity in COVID-19 symptomatic cohorts included in the study (**Supplementary Table 1**). Drug toxicity and adverse reaction to drug are likely associated with the patient’s response to drugs, which were supplied to all symptomatic patients (**Supplementary Table 1**). Hyperlipidemia but not complement deficiency disease correlated with clinical conditions in COVID-19 cohorts (**Supplementary Table 1**). Inflammation is a common condition in COVID-19 patients with increasing symptomatology with disease severity (**Figure 1**). Several of these disorders and COVID-19 disease severity are associated with positive regulation of interleukins (e.g., IL-6) (56) (**Supplementary Figure 5A**, **Supplementary Data 2**). However, in this study, we did not identify interleukins in the serum proteomics dataset, likely due to low protein levels in healthy and asymptomatic cases and interventions to control the so-called “cytokine storm” in symptomatic COVID-19 patients (**Supplementary Figure 7**). Other identified diseases such as amyloidosis, complement deficiency disease, age-related macular degeneration, and glycogen storage disease type II have not been previously directly associated with COVID-19 at least as evidenced in this study. These diseases and conditions may be used to monitor risk factors for COVID-19 disease symptomatology.

## Discussion

In this study, SWATH-MS quantitative serum proteomics together with multiple data analysis algorithms was used to characterize host response to SARS-CoV-2 infection in different cohorts from asymptomatic individuals to severe patients. Due to the complexity of COVID-19 symptomatology, this approach contributed to a better understanding of disease mechanisms and symptoms with possible medical complications at different levels and the identification of potential diagnostic/prognostic biomarkers and drug targets (22–25). The results corroborated previous findings in COVID-19 studies and highlighted how the representation of dysregulated serum proteins and associated BPs increases with COVID-19 disease symptomatology from asymptomatic to severe cases (4–6, 8–13, 18, 22–25). However, the analysis was focused on results that provided new insights into COVID-19 disease symptomatology and potential biomarker proteins for diagnostic and therapeutic interventions (**Figure 7**).

Of the significantly dysregulated proteins, selected immune-related proteins PZP, SELENOP, PON1, and CBP2 were validated as candidate prognostic biomarkers for COVID-19 symptomatology (**Table 1** and **Figure 7**). Of them, PZP was underrepresented in asymptomatic cases only. This protein is a broad-spectrum immunosuppressive protein that suppresses T-cell function during pregnancy to prevent fetal rejection, and its overrepresentation correlates with airway infection and bronchiectasis disease severity (50). Consequently, serum PZP protein levels may be used as a biomarker for COVID-19 disease symptomatology and prognosis of asymptomatic carriers. Selenoprotein levels related to selenium (Se) status affect immune defense and tissue homeostasis through its effect on the trafficking of tissue macrophages (57, 58), and thus SELENOP may be used as a biomarker for disease recovery. PONs have the capacity to protect cells from oxidative stress and are implicated in the pathogenesis of inflammatory diseases (59, 60). Findings suggest a role for PON1 against atherosclerosis and obesity and protective capacity against bacterial, parasitic, and viral infectious diseases (59). Regarding COVID-19, PON1 has been shown to increase in protein levels from nonsevere to severe patients (22) and we found the protein underrepresented in nonsevere and recovered patients, thus suggesting a biomarker for disease recovery. CPB2 appears to have a role in innate immunity through inactivation of complement component C5a, which can induce inflammatory pathways *via* C5aR receptor (41, 61). In our study, CPB2 was overrepresented in all but asymptomatic COVID-19 patients, thus providing a candidate biomarker for disease severity. As expected, the serum levels of these biomarkers correlated with the anti-SARS-CoV-2 Spike IgG levels previously shown to significantly increase from asymptomatic to severe cohorts included in this study (28).

Enrichment analyses were used to identify prognostic biomarker proteins and association to other human diseases and conditions (**Figure 7** and **Supplementary Figure 6**). The BP enrichment and association analyses showed that network representation of nuclear receptor subfamily 1, group H, member 4 (NR1H4) target genes increased with COVID-19 disease severity (**Figure 7**). The farnesoid X receptor (FXR, NR1H4) encodes a ligand-activated transcription factor, which shares structural features in common with nuclear hormone receptor family that functions as a receptor for bile acids (BA) and regulation of the expression of genes involved in bile acid synthesis and transport, lipid and glucose homeostasis, and innate immune and inflammatory responses (62). NR1H4 is essential for BA homeostasis while FXR and its hepatic and intestinal target genes transcriptionally regulate BA synthesis, detoxification, secretion, and absorption in the enterohepatic circulation. Furthermore, FXR agonists as well as a fibroblast growth factor 19 (FGF19) analogue are currently tested in clinical trials for different cholestatic liver diseases (57). The FOXA1 transcription factor network (HNF3A pathway M285) BP with overrepresented proteins in response to COVID-19 increased in enrichment with disease severity (**Figure 7**). This pathway (https://www.gsea-msigdb.org/gsea/msigdb/cards/PID_HNF3A_PATHWAY) coordinates function of primary airway epithelial cells (63) and has been associated with more aggressive breast (64) and prostate cancer (65). Accordingly, considering disorders and processes associated with COVID-19, these proteins may be proposed as candidate prognosis biomarkers for disease progression and severity (**Figure 7**).

The network of interactions between proteins and BPs characterized using Graph Theory algorithms reflected patterns in correlation with COVID-19 disease severity (**Figure 7** and **Supplementary Figure 2**). A distinctive finding using this approach was the acute-phase response SAA2–SAA4 (SAA2–4) readthrough proteins, whose overrepresentation was associated with nonsevere and severe COVID-19 patients (**Figure 7**). The SAA2 has been used to monitor the severity of COVID-19 and as a biomarker for SARS-CoV-2 infection (4). However, the increase in the expression of SAA2–4 coding acute-phase reactant genes or serum protein levels has not been directly associated with COVID-19 patients but with clear cell renal carcinoma (66) and lung cells (67). Therefore, these proteins constitute biomarkers for SARS-CoV-2 infection and prognosis of disease severity (**Figure 7**).

Other novel prognostic biomarker proteins related to COVID-19 disease symptomatology were identified (**Table 1** and **Figure 7**) (22, 68). These biomarkers included potential prognostic tools for SARS-CoV-2 infection, disease symptomatology, progression and recovery, and reduction in thyroiditis. To contribute to the application of these findings in the clinic, some of these prognostic biomarkers were validated using protein-specific ELISA tests (**Figure 7**) and could be incorporated into the daily routine for disease diagnosis/prognosis. Recently, the glycoprotein Galectin-9 (Gal-9) involved in innate immunity and associated with cytokine release syndrome was identified as a surrogate diagnostic biomarker in SARS-CoV-2 infection (69). In our proteomics study, Gal-9 was not identified, but in accordance with these results, the Galectin-3-binding protein (Gal-3BP) with a role in innate immune response to viruses (70) was significantly overrepresented in all symptomatic COVID-19 cohorts (**Supplementary Data 1**).

At the level of other human diseases and conditions, findings revealed potential disorders associated with COVID-19 (**Figure 7** and **Supplementary Figure 6**). Hyperlipidemia and other forms of dyslipidemia have been associated with COVID-19 severity (71) and may be related to FXR and NR1H4 BP enrichment. Amyloidosis in its different forms is caused by deposition of immunoglobulin light chains and have not been previously associated with COVID-19 except for the management of patients with this condition (72). Accordingly, immunoglobulin lambda and kappa variable light chains were overrepresented in nonsevere and severe patients when compared to healthy individuals (**Figures 3A, C**). Another interesting finding was the complement deficiency disease (**Figure 7**). The complement cascade that is directly associated with blood coagulation alterations (73, 74) has been implicated in COVID-19 pathology (**Supplementary Figure 4**) (75). However, in our study, complement, coagulation, and clotting cascades were clearly directly associated with COVID-19 severity, which may explain the association with complement deficiency disease and thrombosis disorders. One of the pathologies identified in our analysis was the age-related macular degeneration (**Figure 7**). This pathology is directly associated with dysregulation of complement regulators such as factor H, which is treated with these factors as therapeutic interventions (76) and has not been associated with COVID-19 but with the primary systemic amyloidosis identified here as enriched with disease severity (**Figure 7**) (77). Another pathology identified as a correlate of disease severity was glycogen storage disease type II, a lysosomal disease not previously related to COVID-19. The immunity to glycan Galα1-3Galβ1-(3)4GlcNAc-R (α-Gal), which was recently related to tick bites and allergic reactions to mammalian meat consumption (alpha-gal syndrome) (78, 79), has been implicated in the protective response to COVID-19 (28, 80). Complement component C3 and hemoglobin subunit beta (HBB) were associated with the immune response to α-Gal in the zebrafish animal model (81) and were both significantly overrepresented in COVID-19 patients when compared to healthy individuals (**Supplementary Data 1**). In humans, the endogenous source of α-Gal is gut bacteria (78), and glycan metabolism has a key role in shaping microbiota composition (82). Therefore, the dysregulation in C3 and HBB serum protein levels observed in COVID-19 cohorts and previously reported in response to α-Gal51 may be due to gut microbiota dysbiosis associated to SARS-CoV-2 infection and COVID-19 severity (83, 84) (**Supplementary Figure 8**). Apolipoprotein A (APOA) isoforms A-I, A-II, and A-IV were significantly dysregulated in COVID-19 patients and serum protein levels decreased with disease symptomatology (**Figure 4** and **Supplementary Data 1**). Lipoprotein(a)-containing APOAs are endogenous triggers of innate immunity and can induce trained immunity (TRIM) (85), thus suggesting that TRIM associated with bacillus Calmette-Guérin (BCG) vaccination may be affected in COVID-19 patients (86, 87) (**Supplementary Figure 8**). Altogether, these disorders and physiological processes should be considered to improve monitoring of COVID-19 symptomatology and as potential targets for therapeutic interventions to reduce the risk for severe symptoms and mortality (23, 24).

A better understanding of COVID-19 on human molecular pathophysiology is required for the identification of new biomarkers and diagnostic and therapeutic targets. By August 9, 2021, 56 publications appear in PubMed with search keywords “covid AND serum AND proteomic” (https://pubmed.ncbi.nlm.nih.gov/?term=covid+serum+proteomic&sort=date). These publications confirmed previous results in studies with different cohorts, populations, and settings and/or provided new serum biomarkers related to disease progression and symptomatology. For example, among the latest publications on this list, Pavel et al. (88) confirmed the association between Th2/Th1 cytokine imbalance and COVID-19 risk mortality; Singh et al. (89) confirmed the increase in serum inflammatory markers in COVID-19 patients; Mitamura et al. (90) confirmed cytokine storm in severe COVID-19 patients; Lazari et al. (91) confirmed and validated SAA1 and SAA2 proteins as biomarkers in low- and high-risk COVID-19 patients; Völlmy et al. (92) proposed various serum proteins as biomarkers to predict mortality in COVID-19 patients; Geyer et al. (93) showed a functional association between serum proteins, biological processes, and clinical parameters between COVID-19 patients and symptomatic but PCR-negative individuals; Laudanski et al. (94) identified serum proteins with potential role in COVID-19 pathology; and Gutmann et al. (95) found mannose binding lectin 2 and pentraxin-3 (PTX3) of the innate immune system as positively associated with COVID-19 mortality.

Our study is the first to provide serum proteomic profiles of cohorts of SARS-CoV-2-infected recovered (hospital discharge), nonsevere (hospitalized), and severe (ICU) cases with increasing systemic inflammation in comparison with healthy individuals sampled prior to the COVID-19 pandemic. The results not only confirmed previous results but provided new serum biomarkers, BPs, and physiological disorders related to disease progression and symptomatology (**Figure 7**). The confirmation of previous results in studies conducted with different cohorts and populations as shown here for the first time in Spain is important to validate diagnostic and therapeutic interventions at a global scale affecting this pandemic. The new prognostic biomarkers associated with COVID-19 reported here not only serve in conjunction with diagnostic RNA, antigen, and antibody detection tests to complement other previously identified biomarkers such as IL-6, but also provide the possibility of using highly abundant serum proteins for prognosis of disease severity (e.g., CBP2, up to 0.1 mg/ml), asymptomatic carriers (e.g., PZP, up to 350 ng/ml), or disease recovery (e.g., PON1, up to 160 ng/ml). The disorders and processes associated with the new biomarkers identified in this study provide clinical tools for the evaluation and treatment of SARS-CoV-2 infection and disease symptomatology and progression (**Figure 7**). For example, detection of high HNF3A levels in nonsevere or severe patients suggests their diagnosis and treatment to reduce airway dilatation with production of large cysts associated with function of airway epithelial cells (96).

The main limitations of this study include the following (a) possible effect on serum protein representation of immunosuppressive treatments to control the cytokine storm in symptomatic COVID-19 patients (**Supplementary Table 1**); (b) impact of comorbidities associated or not to COVID-19 (**Supplementary Table 1**); (c) serum samples were collected when the main circulating SARS-CoV-2 variant was WIV04/2019 and thus possible differences with other variants in the serum protein response to infection and caused pathologies should be considered; (d) although serum proteomics analysis was conducted with samples on each cohort including three pools of 5–10 individuals each (**Figure 1**), studies with a larger number of samples and/or on individual cases may provide case-by-case differences in serum protein representation; and (e) as samples were collected from a retrospective study (28), the effect of some factors such as age (oldest in nonsevere cases; **Figure 1**) but not sex ratio (similar in all groups; **Figure 1**) may affect protein representation. However, because age did not show significant differences between severe and asymptomatic or heathy cohorts, possible differences in age-related serum protein representation should not affect the main results of the study.

In conclusion and despite these limitations, the SWATH-MS quantitative serum proteomics used in our study together with multiple data analysis algorithms contributed to the characterization of SARS-CoV-2–host molecular interactions and advanced translational medicine by identifying prognostic biomarker proteins and physiological disorders with potential implications for disease diagnosis/prognosis contributing to the control of the COVID-19 pandemic. The identified biomarkers for disease recovery (SELENOP and PON1), severity (CBP2), and symptomatology (PZP) could be used for disease prognosis. For example, in some cases, hospitalized nonsevere patients could progress to disease recovery (hospital discharge) or severity (ICU). In our study, the results showed that some of these biomarkers may be used to evaluate the risk of hospitalized patients to develop severe symptoms.

## Data Availability Statement

The mass spectrometry proteomics data have been deposited to the ProteomeXchange Consortium via the PRIDE partner repository with the dataset identifier PXD024549 and 10.6019/PXD024549. The datasets presented in this study can be found in online repositories. The names of the repository/repositories and accession number(s) can be found in the article/**Supplementary Material**.

## Ethics Statement

The studies involving human participants were reviewed and approved by Ethical and Scientific Committees (University Hospital of Ciudad Real C-352 and SESCAM C-73). Written informed consent for participation was not required for this study in accordance with the national legislation and the institutional requirements.

## Author Contributions

JF, JU, MV, and CG designed the study. MV, SA-J, EF-C, MC, and IGF performed the experiments. MV, JF, JU, FR-d-R, NJ-C, and AE-P collected and analyzed data. JF, CG, JU, IGF, and MV supervised the project. All authors wrote the manuscript. All authors contributed to the article and approved the submitted version.

## Funding

This research was partially funded by Junta de Comunidades de Castilla-La Mancha (JCCM), Spain and EU-FEDER (grants MYCOTRAINING SBPLY/19/180501/000174 and GALINFEC SBPLY/17/180501/000185). EF-C has a predoctoral grant from UCLM. MC was funded by the Ministerio de Ciencia, Innovación y Universidades, Spain (grant FJC-2018-038277-I). IGF was supported by UCLM. MV was supported by UCLM and EU-FEDER.

## Conflict of Interest

The authors declare that the research was conducted in the absence of any commercial or financial relationships that could be construed as a potential conflict of interest.

## Publisher’s Note

All claims expressed in this article are solely those of the authors and do not necessarily represent those of their affiliated organizations, or those of the publisher, the editors and the reviewers. Any product that may be evaluated in this article, or claim that may be made by its manufacturer, is not guaranteed or endorsed by the publisher.

## Supplementary Material

Supplementary Figure 1 Analysis of differentially represented proteins in response to COVID-19.

Supplementary Figure 2 High-resolution network analysis of interactions between proteins and BPs characterized using Graph Theory algorithms.

Supplementary Figure 3 Serum proteomics data for previously identified biomarkers for COVID-19 disease severity.

Supplementary Figure 4 Metascape analysis of differentially represented proteins and previously reported in COVID-19 omics datasets.

Supplementary Figure 5 Differential representation of proteins not previously reported in COVID-19 omics datasets.

Supplementary Figure 6 Association of differentially represented proteins in response to COVID-19 to other human diseases and conditions.

Supplementary Figure 7 Cytokine (IL-1 and IL-4) response in COVID-19 symptomatic patients and healthy controls.

Supplementary Figure 8 Mechanisms of potential disorders associated with COVID-19.

Supplementary Table 1 Clinical parameters and comorbidities in COVID-19 asymptomatic and symptomatic cohorts.

Supplementary Data Sheet 1 Serum proteomics analysis.

Supplementary Data Sheet 2 Network analysis of interactions between proteins and BPs characterized using Graph Theory algorithms. Please see the Supplementary Material section of the Author guidelines for details on the different file types accepted.
