# Supplementary material for: Characterization by Quantitative Serum Proteomics of Immune-Related Prognostic Biomarkers for COVID-19 Symptomatology
Source: Front Immunol. 2021 Sep 8;12:730710. doi: 10.3389/fimmu.2021.730710 (PMC8457011; doi:10.3389/fimmu.2021.730710)
Supplement: Supplementary Figure 1 — Analysis of differentially represented proteins in response to COVID-19. [file DataSheet_1.pdf]

Characterization by quantitative serum proteomics of immune-related prognostic biomarkers for COVID-19 symptomatology

Margarita Villar, José Miguel Urra, Francisco J. Rodríguez-del-Río, Sara Artigas-Jerónimo, Natalia Jiménez-Collados, Elisa Ferreras-Colino, Marinela Contreras, Isabel G. Fernández de Mera, Agustín Estrada-Peña, Christian Gortázar, José de la Fuente

Additional information

Supplementary Fig. 1. Analysis of differentially represented proteins in response to COVID-19.
Supplementary Fig. 2. High-resolution network analysis of interactions between proteins and BPs characterized using Graph Theory algorithms.
Supplementary Fig. 3. Serum proteomics data for previously identified biomarkers for COVID-19 disease severity.
Supplementary Fig. 4. Metascape analysis of differentially represented proteins and previously reported in COVID-19 omics datasets.
Supplementary Fig. 5. Differential representation of proteins not previously reported in COVID-19 omics datasets.
Supplementary Fig. 6. Association of differentially represented proteins in response to COVID-19 to other human diseases and conditions.
Supplementary Fig. 7. Cytokine (IL-1 and IL-4) response in COVID-19 symptomatic patients and healthy controls.
Supplementary Fig. 8. Mechanisms of potential disorders associated with COVID-19.
Supplementary Table 1. Clinical parameters and comorbidities in COVID-19 asymptomatic and symptomatic cohorts.

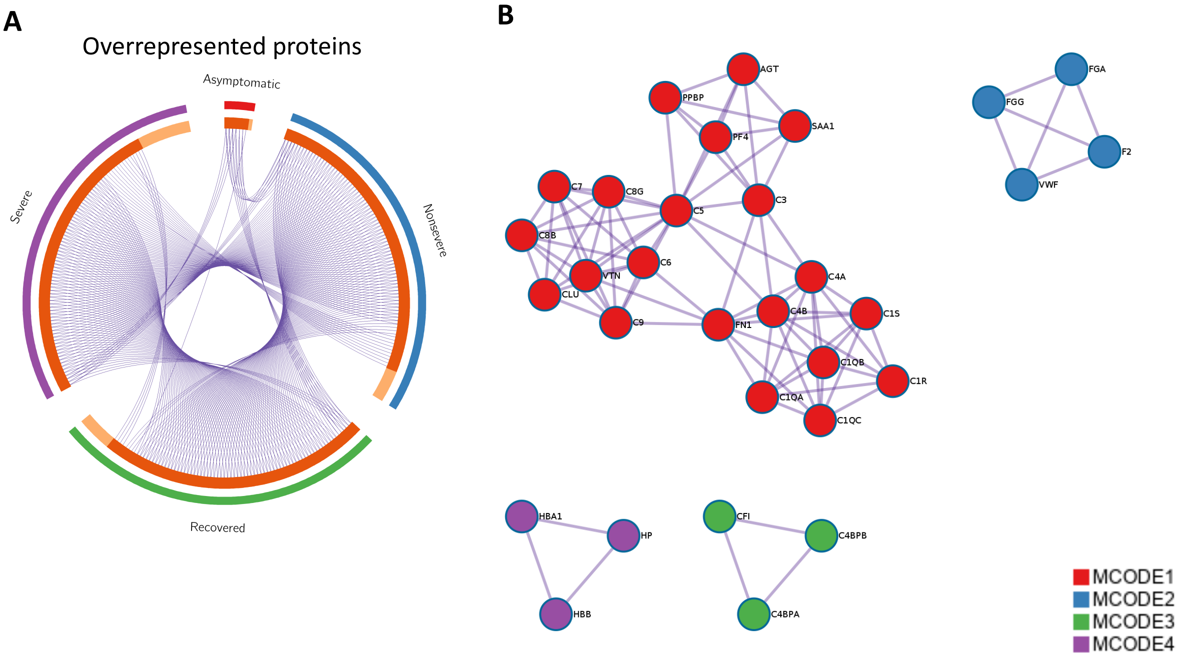

All lists merged Colored by Cluster(Keep MCODE Nodes Only)

Color	MCODE	GO	Description	Log10(P)
	MCODE_1	R-HSA-977606	Regulation of Complement cascade	-41.3
	MCODE_1	R-HSA-166658	Complement cascade	-39.6
	MCODE_1	ko04610	Complement and coagulation cascades	-37.2
	MCODE_2	GO:0072378	blood coagulation, fibrin clot formation	-12.1
	MCODE_2	R-HSA-140877	Formation of Fibrin Clot (Clotting Cascade)	-11.5
	MCODE_2	R-HSA-76009	Platelet Aggregation (Plug Formation)	-11.5
	MCODE_3	R-HSA-977606	Regulation of Complement cascade	-8.4
	MCODE_3	R-HSA-166658	Complement cascade	-8.1
	MCODE_3	ko04610	Complement and coagulation cascades	-7.7
	MCODE_4	R-HSA-2168880	Scavenging of heme from plasma	-10.1
	MCODE_4	GO:0042744	hydrogen peroxide catabolic process	-8.9
	MCODE_4	R-HSA-2173782	Binding and Uptake of Ligands by Scavenger Receptors	-8.5

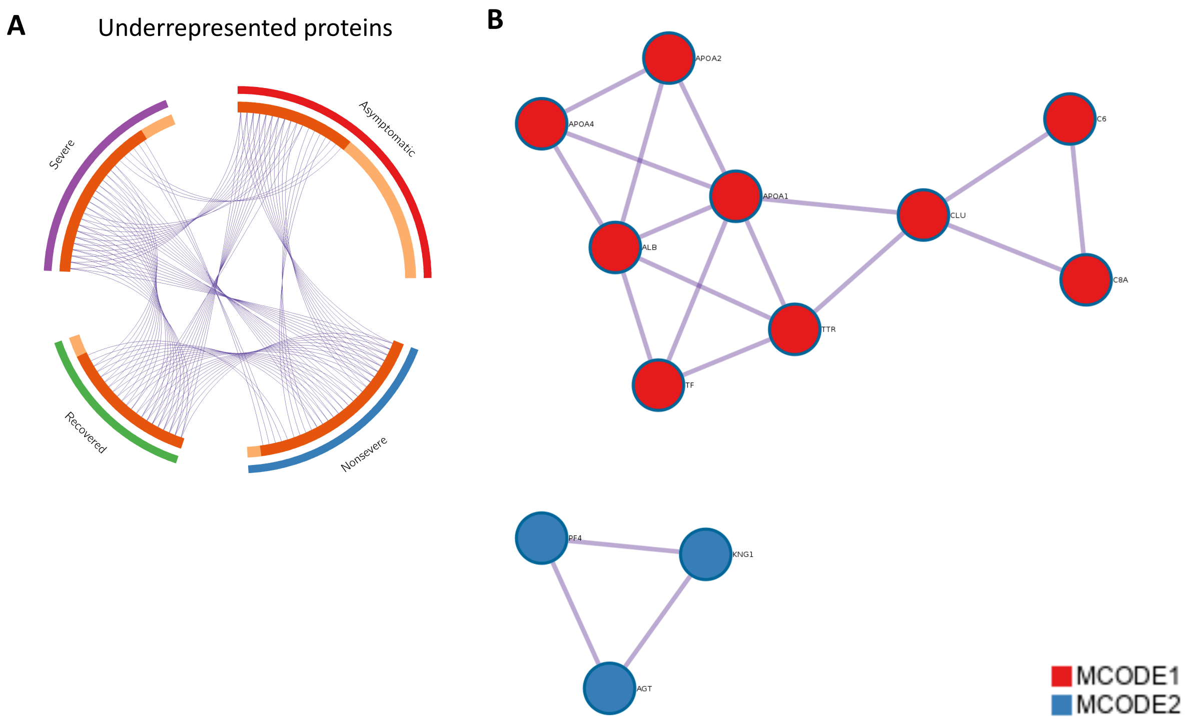

All lists merged Colored by Cluster(Keep MCODE Nodes Only)

Color	MCODE	GO	Description	Log10(P)
	MCODE_1	GO:0034375	high-density lipoprotein particle remodeling	-10.8
	MCODE_1	GO:0043691	reverse cholesterol transport	-10.5
	MCODE_1	GO:0034369	plasma lipoprotein particle remodeling	-9.8
	MCODE_2	R-HSA-375276	Peptide ligand-binding receptors	-6.4
	MCODE_2	R-HSA-373076	Class A/1 (Rhodopsin-like receptors)	-5.8
	MCODE_2	R-HSA-418594	G alpha (i) signalling events	-5.5

Supplementary Fig. 1. Analysis of differentially represented proteins in response to COVID-19. Data was separately analyzed for overrepresented and underrepresented proteins using the Metascape gene annotation and analysis resource (https://metascape.org/gp/index.html#/main/step1). (A) Ciscos plot of the overlap between protein lists. On the outside, each arc represents the identity of each protein list. On the inside, each arc represents a protein list, where each protein has a spot on the arc. Dark orange color

represents the proteins that appear in multiple lists and light orange color represents proteins that are unique to that protein list. Purple lines link the same protein that are shared by multiple protein lists. The greater the number of purple links and the longer the dark orange arcs imply greater overlap among the input protein lists. (B) Protein-protein interaction enrichment analysis. For each protein list, protein-protein interaction enrichment analysis was carried out with the BioGrid, InWeb_IM and OmniPath databases. The resultant network contains the subset of proteins that form physical interactions with at least one other member in the list. If the network contains between 3 and 500 proteins, the Molecular Complex Detection (MCODE) algorithm was applied to identify densely connected network components. The MCODE networks identified for individual protein lists were gathered and shown in the table. All lists merged colored by cluster keeping MCODE nodes only.

A

Healthy

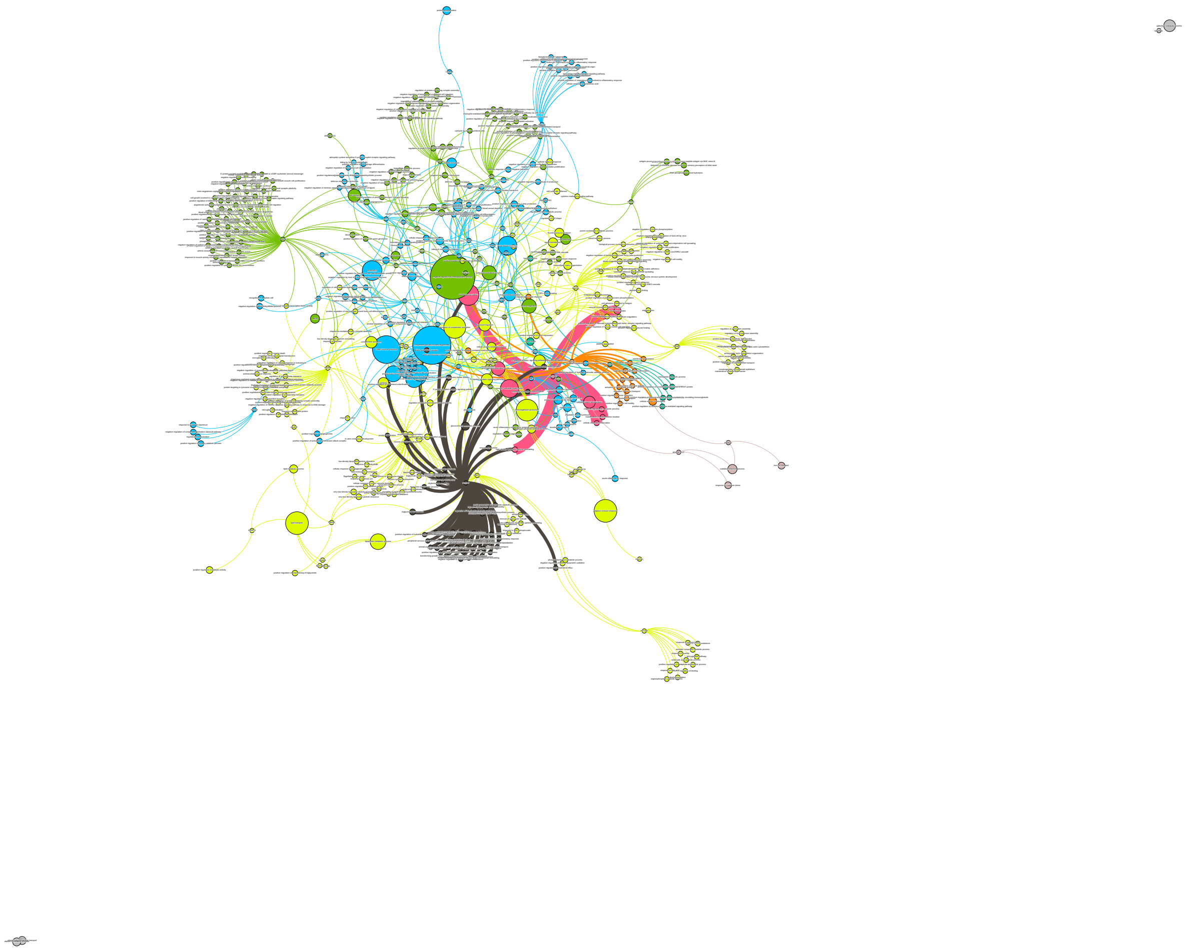

B

Asymptomatic

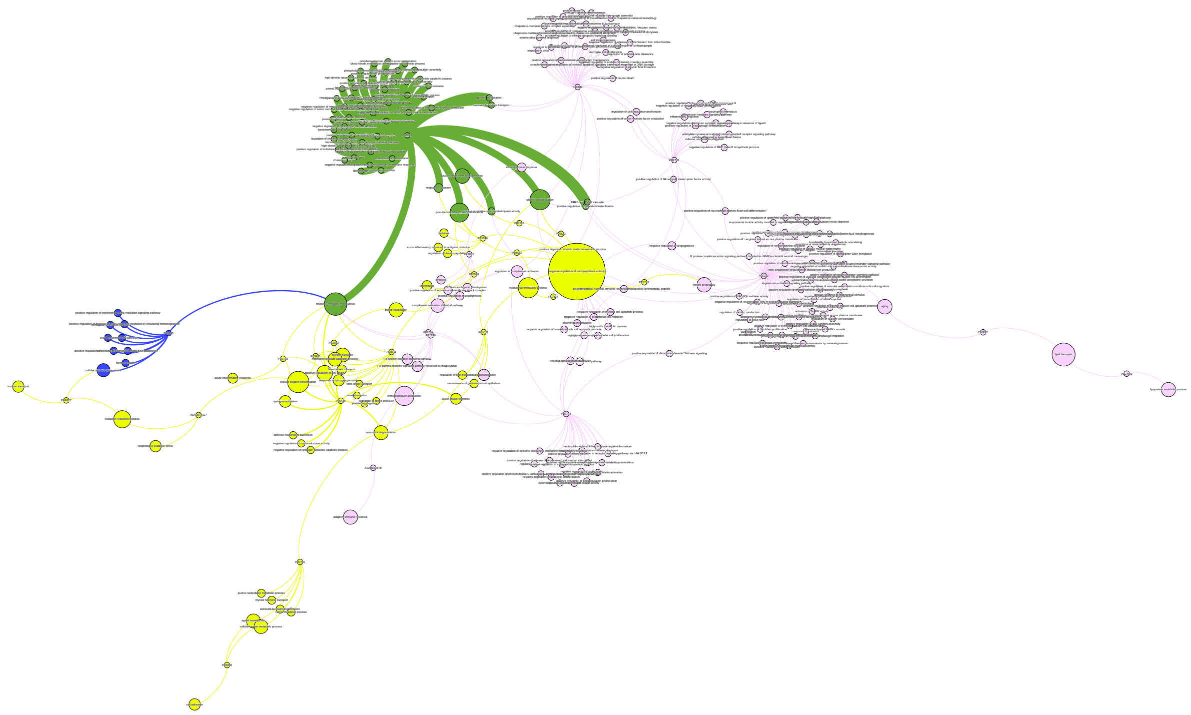

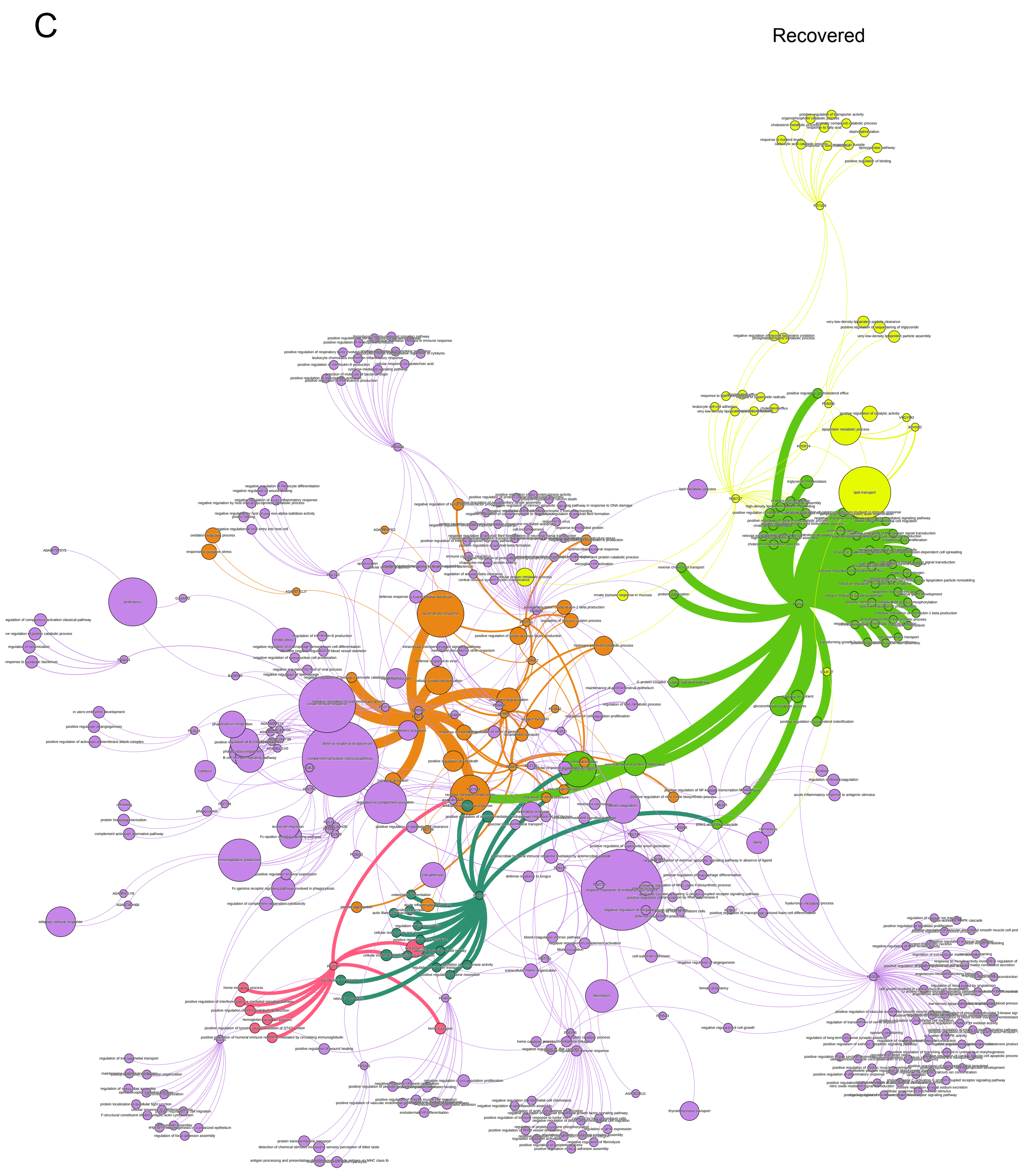
C
Recovered

D

Nonsevere

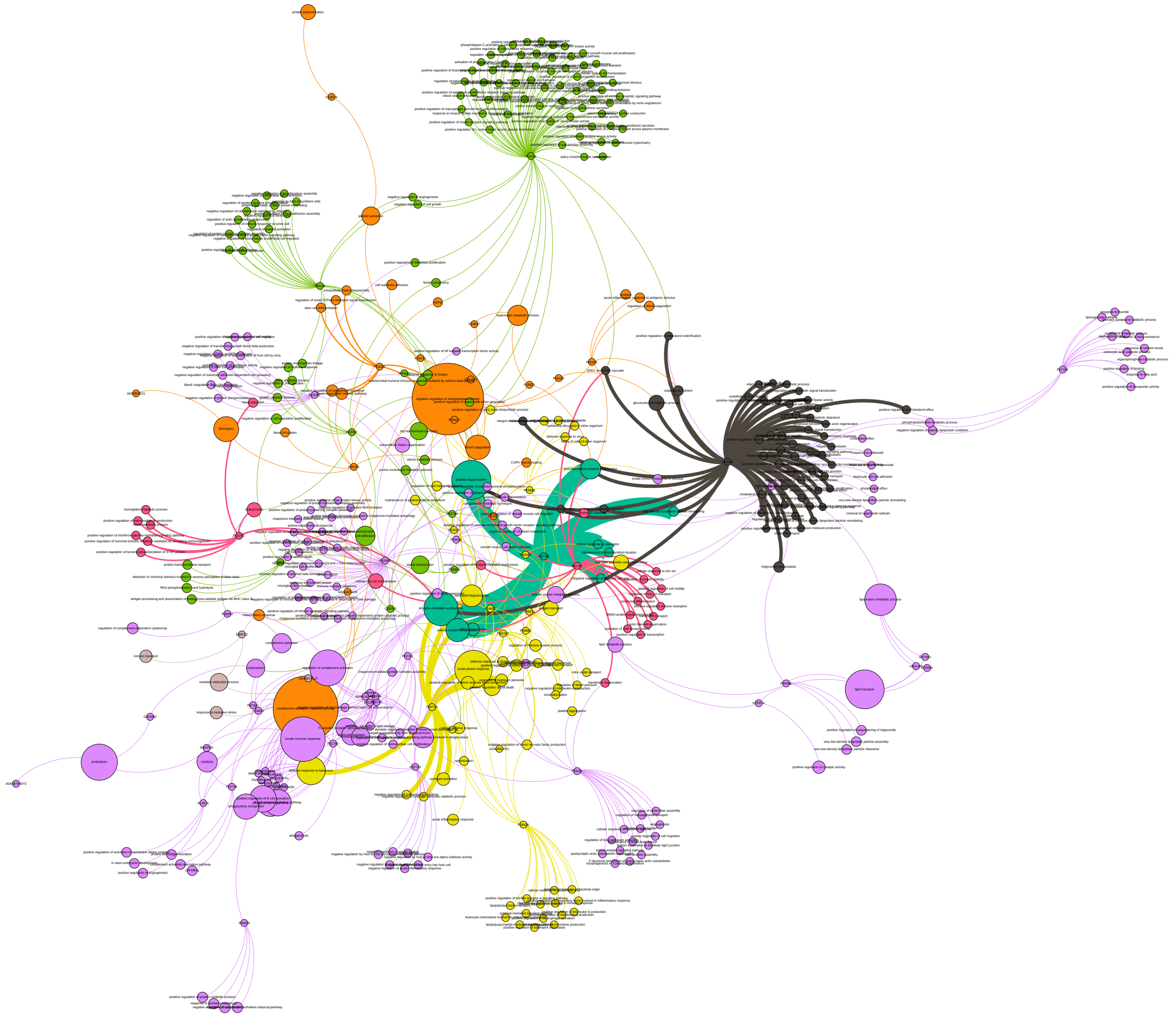

E

Severe

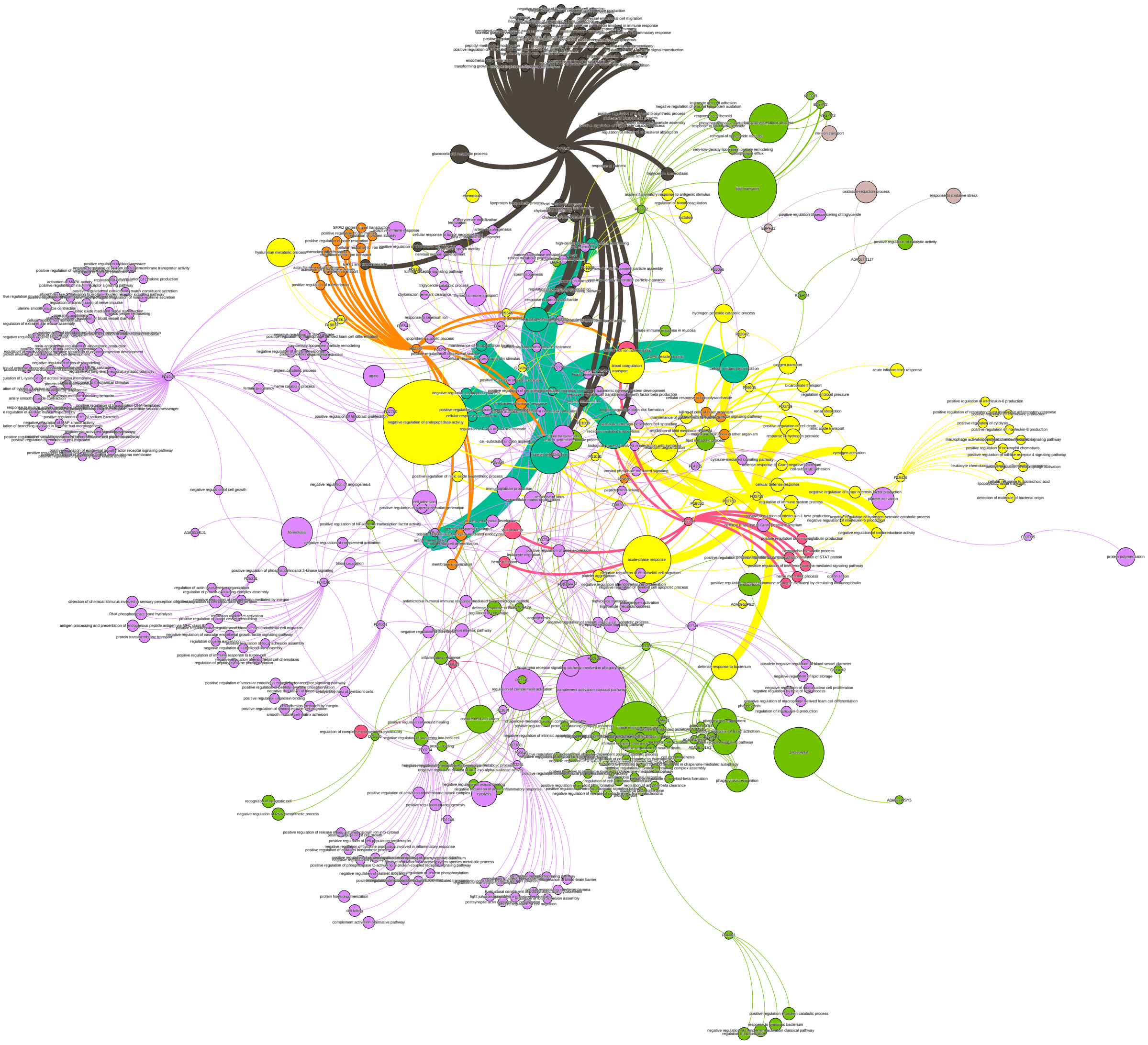

Supplementary Fig. 2. High-resolution network analysis of interactions between proteins and BPs characterized using Graph Theory algorithms. High resolution networks reflect the proteins and BPs in (A) healthy, (B) asymptomatic, (C) recovered, (D) nonsevere and (E) severe individual cohorts. The colors of the chart show cluster, or groups of proteins and BPs

that interact more frequently among them than with other nodes. Colors are random and each color only shows the cluster (i.e., colors among networks are not comparable). Size of each node indicates its relative importance in the network (the larger, the higher). Links have the same color as the cluster they belong to.

Protein	Healthy	Asymptomatic	Recovered	Nonsevere	Severe
SAA1	10184	7207	94778	559502	1949760
SAA2	4700	3743	18128	81063	323900
CRP	5658	5696	19905	61939	195869
GSN	226277	219667	150933	143252	79405
SERPINA1	62056	27454	82904	65861	60060
SERPINA3	782285	655369	1959442	2908944	5367289
SERPINA4	27889	23040	29142	24936	29456
SERPINA6	99436	72879	83268	85565	78841
SERPINA7	15690	17400	24326	29864	22497
SERPINA10	1437	1721	2627	2607	3284
SERPINC1	637076	431107	526825	548961	761268
SERPIND1	408883	336719	545786	460745	531274
SERPINF1	74741	68998	96863	110872	144983
SERPINF2	167226	140700	193292	162731	208041
SERPING1	586124	719641	1378754	1615527	1596314

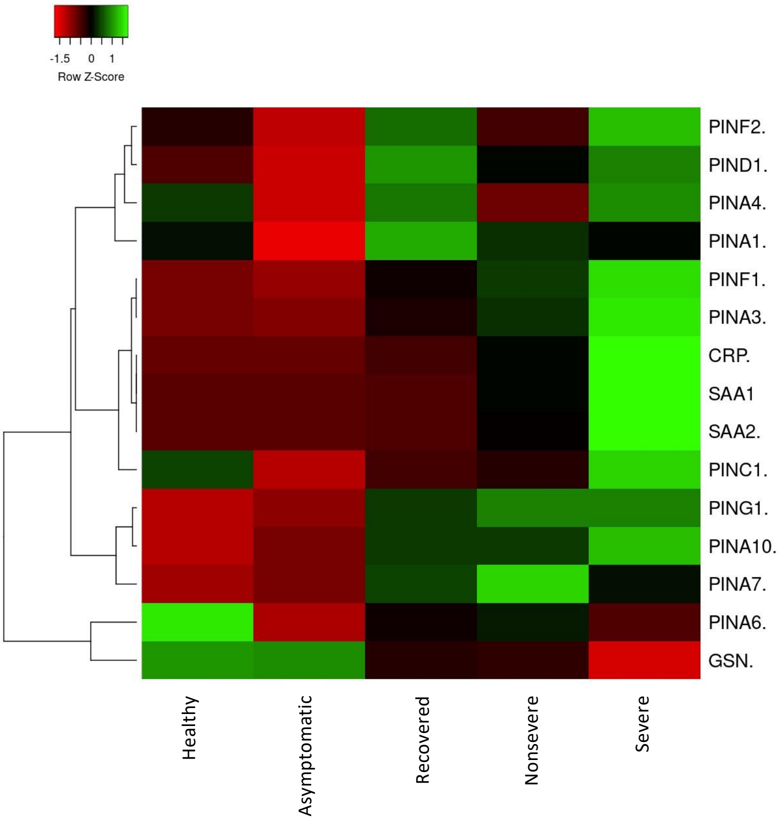

Supplementary Fig. 3. Serum proteomics data for previously identified biomarkers for COVID-19 disease severity. Proteins with significant differences when compared to healthy controls are shown in red (Welch's unpaired t test; $p < 0.05$). SERPINS were abbreviated in the heatmap as PIN. Biomarker's identification was obtained from previous reports.

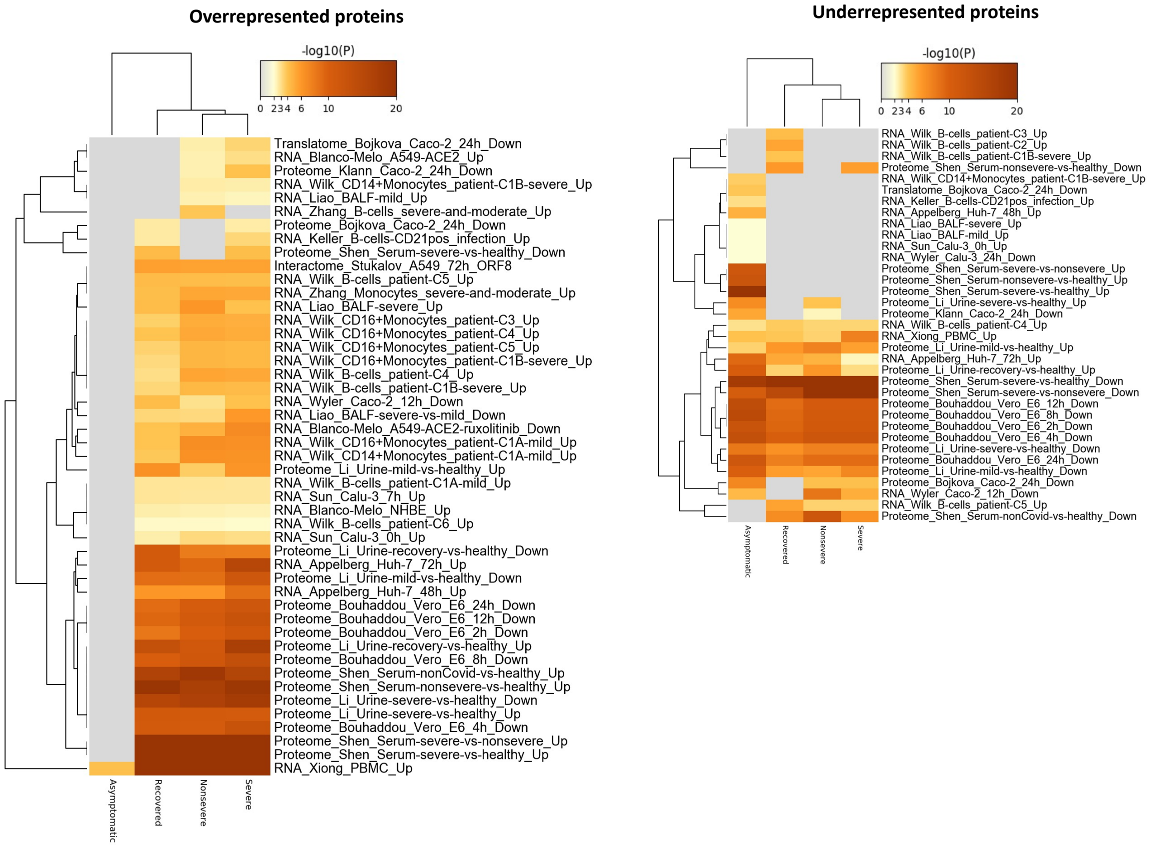

Supplementary Fig. 4. Metascape analysis of differentially represented proteins and previously reported in COVID-19 omics datasets. Enrichment analysis was conducted using the Coronascape COVID database (https://metascape.org/COVID) to identify proteins found in our study as differentially represented in response to COVID-19 and reported in previous COVID-19 omics datasets.

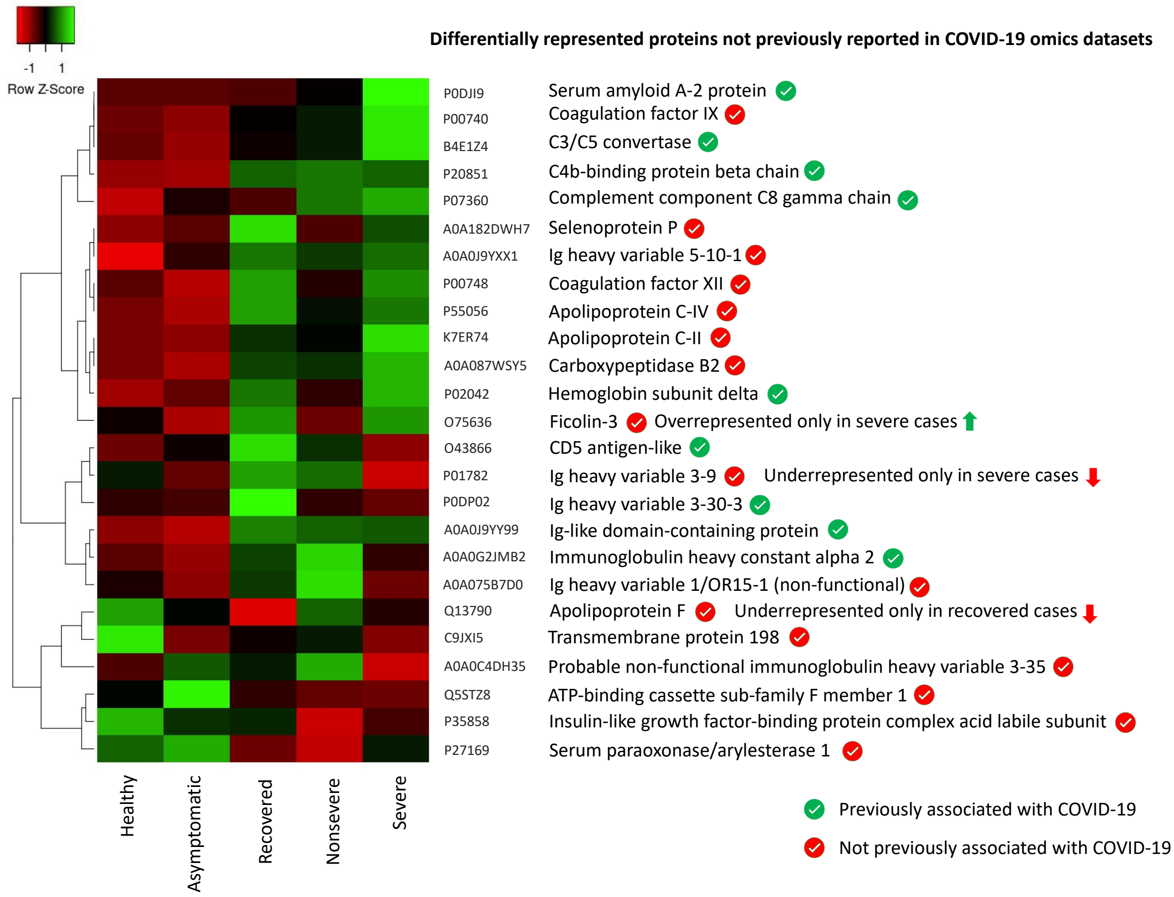

Supplementary Fig. 5. Differential representation of proteins not previously reported in COVID-19 omics datasets. First, an enrichment analysis was conducted using the Coronascape COVID database (https://metascape.org/COVID) to identify proteins found in our study as differentially represented in response to COVID-19 and reported in previous COVID-19 omics datasets. Then, not previously identified differentially represented proteins in response to COVID-19 ($p < 0.05$; unpaired two-sided Welch's t test) were used in the heatmap (Z-scored original value).

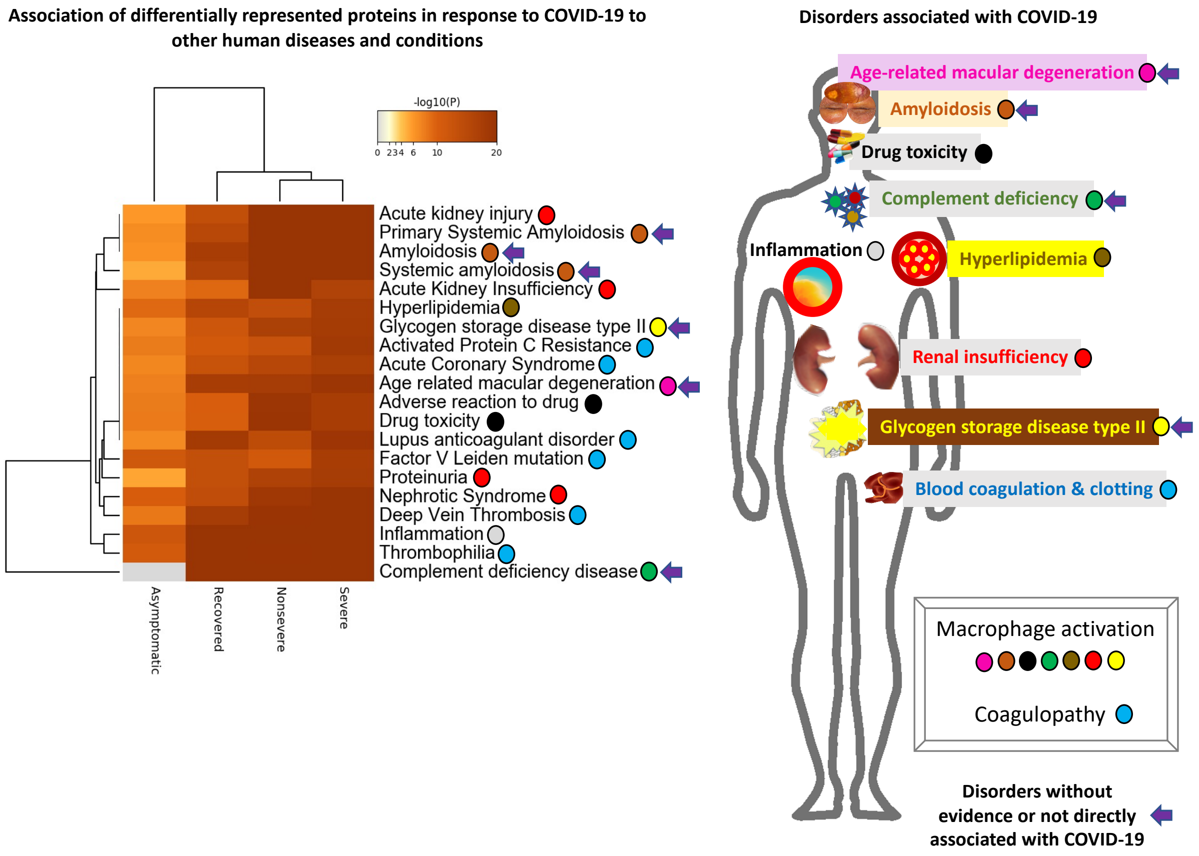

Supplementary Fig. 6. Association of differentially represented proteins in response to COVID-19 to other human diseases and conditions. An enrichment analysis was conducted using the DisGeNET discovery platform (https://www.disgenet.org) to identify proteins differentially represented in response to COVID-19 and associated to other human diseases and conditions. Associated major physiological processes, previous diseases associated with COVID-19 and new findings (purple arrows) are shown.

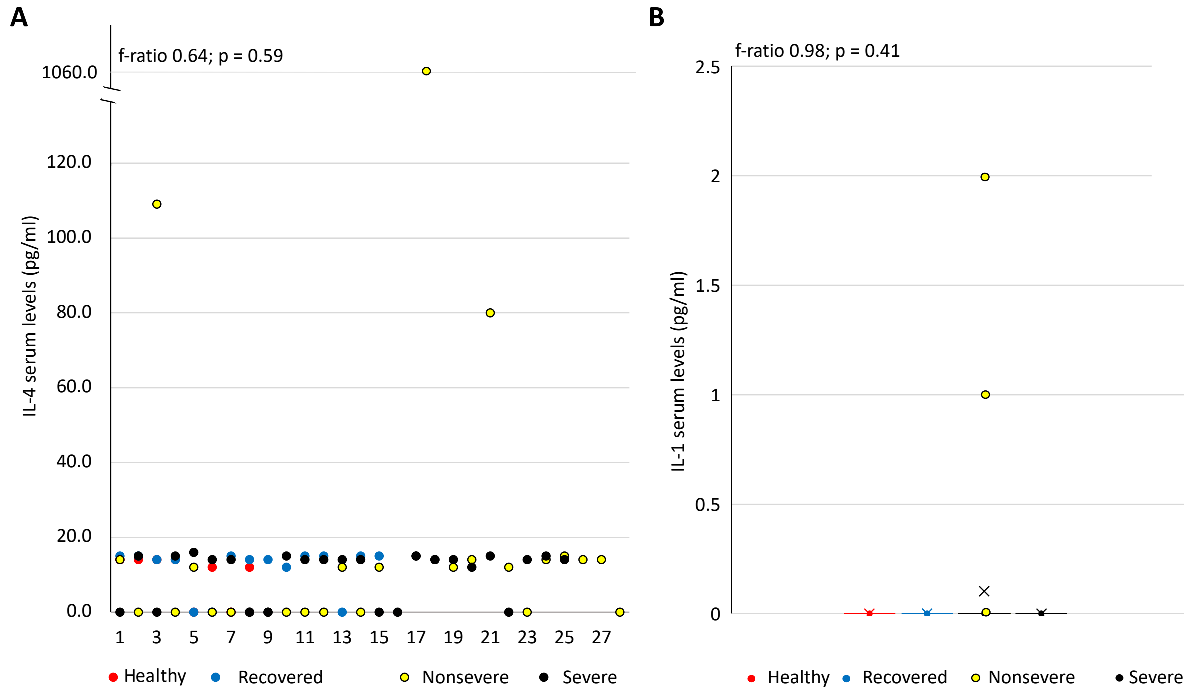

Supplementary Fig. 7. Cytokine (IL-1 and IL-4) response in COVID-19 symptomatic patients and healthy controls. The analysis was focused on (A) anti-inflammatory IL-4 and (B) pro-inflammatory IL-1 serum levels determined by ELISA. Individuals were grouped as healthy controls (n = 37), recovered (n = 27), nonsevere (n = 29) and severe (n = 25). The results were compared between different groups by one-way ANOVA test ($p > 0.05$).

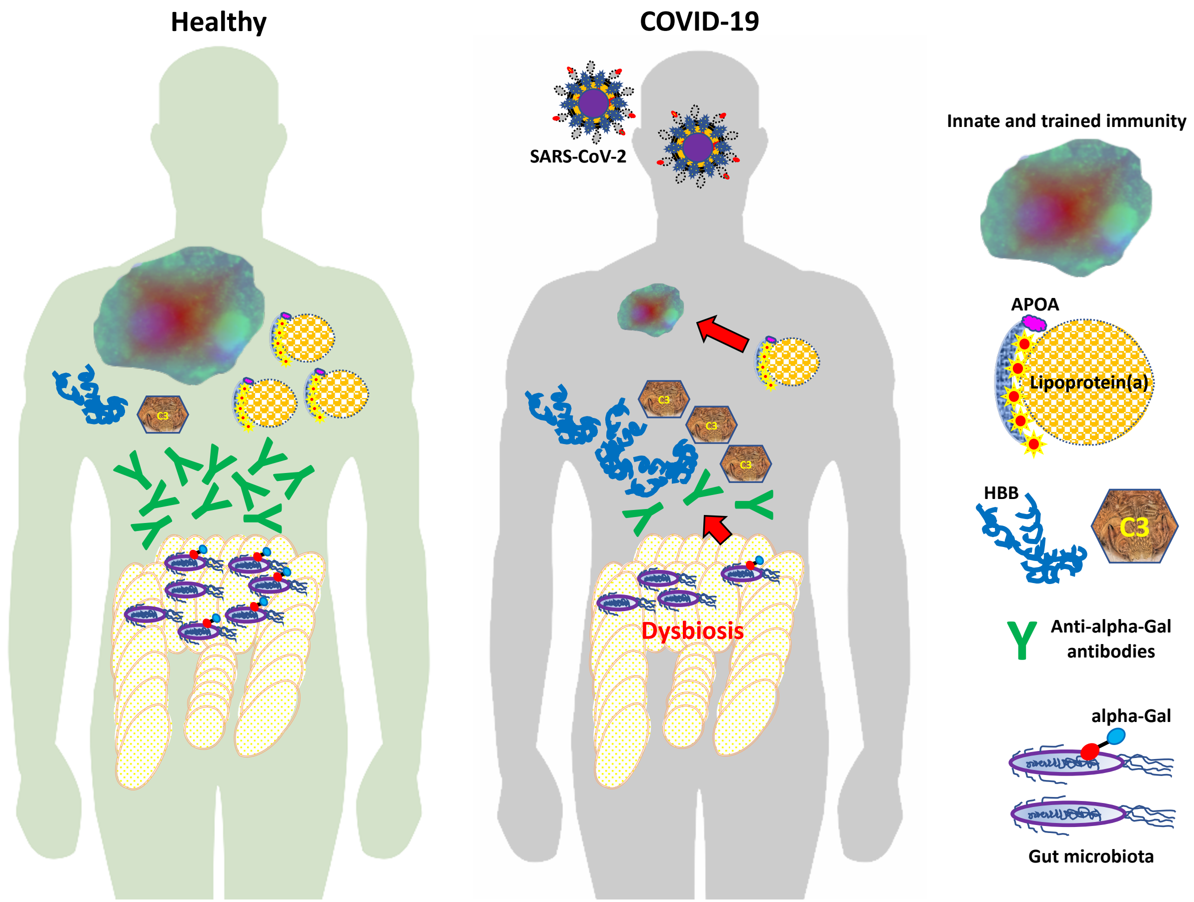

Supplementary Fig. 8. Mechanisms of potential disorders associated with COVID-19. The immunity to glycan alpha-Gal has been implicated in the protective response to COVID-19. Complement component C3 and hemoglobin subunit beta (HBB) have been implicated in the immune response to alpha-Gal and were both significantly overrepresented in COVID-19 patients when compared to healthy individuals. In humans, the endogenous source of alpha-Gal are gut bacteria and glycan metabolism has a key role in shaping microbiota composition. Therefore, the dysregulation in C3 and HBB serum protein levels observed in COVID-19 cohorts as in response to α-Gal may be due to gut microbiota dysbiosis associated to SARS-CoV-2 infection and COVID-19 severity. Apolipoprotein A (APOA) isoforms were significantly underrepresented in COVID-19 and serum protein levels decreased with disease severity. Lipoprotein(a) containing APOAs are endogenous triggers of innate immunity and can induce trained immunity (TRIM), thus suggesting that TRIM may be affected in COVID-19 patients.

Supplementary Table 1. Clinical parameters and comorbidities in COVID-19 asymptomatic and symptomatic cohorts.

**Parameters and comorbidities**	**Asymptomatic**	**Recovered** (hospital discharge)	**Nonsevere** (hospitalized)	**Severe** (ICU)
Neutrophils (10^3 cells/ µl)	Not determined	7.0 ± 4.0	7.7 ± 4.4	14.2 ± 9.6
Neutrophils (%)	Not determined	68.9 ± 14.1	76.8 ± 10.8	85.1 ± 10.9
Lymphocytes (10^3 cells/ µl)	Not determined	1.5 ± 0.5	1.2 ± 0.6	1.1 ± 0.7
Lymphocytes (%)	Not determined	19.0 ± 10.1	13.9 ± 8.4	8.4 ± 7.4
Neutrophil-Lymphocyte Count Ratio (NLR)	Not determined	5.4 ± 4.2	10.1 ± 10.0	18.8 ± 14.8
D-dimer (ng/ml)	Not determined	712 ± 623	1514 ± 1528	6528±9436
C-reactive protein (CRP) (mg/dl)	Not determined	1.0 ± 1.4	4.4 ± 5.7	10.4 ± 9.7
Renal disease (% cases)	0	7	13	6
Obesity (% cases)	11	30	20	33
Lipid alterations (% cases)	22	23	53	33
Diabetes (% cases)	0	7	13	26
Arterial hypertension (% cases)	22	53	53	66
Chronic obstructive pulmonary disease (% cases)	0	7	13	0
Thrombosis (% cases)	0	7	0	0
Coagulation disorder (% cases)	0	0	0	0
Complement alteration (% cases)	0	0	0	0
Immunosuppressive treatment (% cases)	None	Ruxolitinib	Corticosteroids	Acyclovir
Mortality (% cases)	0	0	33	40

COVID-19 asymptomatic and symptomatic cohorts included asymptomatic (n = 16), recovered (n = 27), nonsevere (n = 29), and severe (n = 25) individuals.
